# Enhanced durability of a Zika virus self-amplifying RNA vaccine through combinatorial OX40 and 4-1BB agonism

**DOI:** 10.1172/jci.insight.187405

**Published:** 2025-04-03

**Authors:** Hsueh-Han Lu, Rúbens Prince dos Santos Alves, Qin Hui Li, Luke Eder, Julia Timis, Henry Madany, Kantinan Chuensirikulchai, Krithik V. Varghese, Aditi Singh, Linda Le Tran, Audrey Street, Annie Elong Ngono, Michael Croft, Sujan Shresta

**Affiliations:** 1 Center for Vaccine Innovation, La Jolla Institute for Immunology, La Jolla, California, USA.; 2 Biomedical Sciences Graduate Program, University of California, San Diego, La Jolla, California, USA.; 3 Center for Autoimmunity and Inflammation, La Jolla Institute for Immunology, La Jolla, California, USA.; 4 Department of Pediatrics, School of Medicine, University of California, San Diego, La Jolla, California, USA.

**Keywords:** Immunology, Infectious disease, Vaccines, Costimulation, Mouse models, T cells

## Abstract

The SARS-CoV-2 pandemic highlighted the potential of mRNA vaccines in rapidly responding to emerging pathogens. However, immunity induced by conventional mRNA vaccines wanes quickly, requiring frequent boosters. Self-amplifying RNA (saRNA) vaccines, which extend antigen expression via self-replication, offer a promising strategy to induce more durable immune responses. In this study, we developed an saRNA vaccine encoding Zika virus (ZIKV) membrane and envelope proteins and evaluated its efficacy in mice. A single vaccination elicited strong humoral and cellular immune responses and reduced viral loads but only for 28 days. By day 84, antibody titers and T cell responses had significantly declined, resulting in reduced efficacy. To address this, we evaluated agonist antibodies targeting the T cell costimulatory molecules OX40 and 4-1BB. Coadministration of agonist antibodies enhanced CD8^+^ T cell responses to vaccination, resulting in sustained immunity and reduced viral loads at day 84. Depletion and passive transfer studies verified that long-term antiviral immunity was primarily CD8^+^ T cell dependent, with minimal contributions from antibody responses. These findings suggest that agonists targeting members of the tumor necrosis receptor superfamily, such as OX40 and 4-1BB, might enhance the durability of saRNA vaccine–induced protection, addressing a key limitation of current mRNA vaccine platforms.

## Abstract 2

A self-amplifying RNA vaccine with OX40/4-1BB agonists enhances CD8^+^ T cell responses and long-term protection against Zika virus, improving vaccine durability for emerging pathogens.

## Introduction

The rapid development and deployment of mRNA vaccines played a major role in mitigating the worldwide impact of the COVID-19 pandemic, suggesting that similar strategies could be utilized to combat other emerging pathogens (1). However, it is now clear that conventional mRNA vaccines have limited durability of protection characterized by rapidly waning humoral immunity (2–4), thus necessitating frequent booster doses or updated vaccines to maintain protection. Self-amplifying RNA (saRNA) vaccines represent a significant advance in mRNA vaccine technology and offer the possibility of inducing sustained immunity. Like conventional mRNA vaccines, saRNA vaccines encode antigen-specific mRNA but also include genes encoding viral replicases, which enable rapid amplification of the mRNA. This self-replication machinery is designed to increase and prolong exposure to antigen (5, 6). Sustained antigen expression has the potential to elicit more robust and durable immune responses compared with conventional mRNA vaccines (7, 8) and, as a result, could support single- and low-dose immunization, thereby reducing manufacturing costs, facilitating rapid deployment, and potentially increasing vaccine uptake in the event of future pandemics.

CD4^+^ and CD8^+^ T cell responses play crucial roles in controlling infection and contributing to long-term immunity against viruses (9, 10). Previous work with SARS-CoV-2 in humans has shown that mRNA vaccine–induced T cell immunity tends to persist longer than humoral immunity (11), and early T cell responses to vaccination are associated with faster viral clearance and milder disease in patients with COVID-19 (12–14). Additionally, prior vaccination was shown to elicit faster and stronger immune responses during breakthrough infections, underscoring the importance of durable T cell immunity (15). These findings suggested that mRNA vaccines could be improved by incorporating strategies to further enhance and sustain T cell responses. Several members of the tumor necrosis factor receptor (TNFR) superfamily, including OX40 (CD134) and 4-1BB (CD137), are expressed by T cells and provide costimulatory signals for antigen-specific T cell activation (16). Notably, agonist antibodies (Abs) against these molecules have shown the ability to promote effector and memory T cell responses against a range of targets, including SARS-CoV-2, vaccinia virus, poxvirus, and lymphocytic choriomeningitis virus, as well as various tumors (17–24). Activation of OX40 and 4-1BB can also enhance the development of T follicular helper (Tfh) cells (25, 26), which are crucial for the development of Ab responses to vaccinia virus (27), and the agonist Abs were also shown to synergistically enhance interferon-γ (IFN-γ) production by CD4^+^ and CD8^+^ T cells following poxvirus vaccination (28). Additionally, agonist anti-OX40 and anti–4-1BB Abs improve antitumor CD8^+^ T cell responses in mouse models of melanoma and colon carcinoma (29, 30) and in patients with advanced solid tumors (31). Thus, treatment with agonists of OX40 and 4-1BB, and potentially other costimulatory TNFR members expressed by T cells, represents a potential strategy to boost T cell responses to mRNA vaccines.

Despite advances in vaccine technology and disease epidemiology, safe and effective vaccines are still lacking for a large number of viruses that pose significant threats to global public health because of their potential to cause outbreaks, epidemics, and pandemics (32–34). Although Zika virus (ZIKV) was first isolated in 1947 (35), it was only recognized as a global health threat in 2015, when outbreaks in South America were shown to be associated with devastating neurological consequences, including congenital Zika syndrome in neonates of women infected during pregnancy and Guillain-Barré syndrome in infected adults (36–38). The major 2015–2016 ZIKV outbreak in Brazil resulted in approximately 216,000 infections (39) and 1,500 cases of congenital defects (40). Moreover, the virus continues to circulate at lower levels in many countries (41) and may be accumulating mutations that confer enhanced transmissibility and pathogenicity (42), suggesting that the danger of another major outbreak remains. The urgent need for effective vaccines against ZIKV and other flaviviruses, including dengue virus (DENV), is further emphasized by the facts that these mosquito-borne viruses now cocirculate in many regions (43) and that immunity to one flavivirus has the potential to cross-enhance the severity of disease caused by another flavivirus, further burdening public health programs in many resource-challenged countries (44, 45). Despite intense ongoing efforts, however, there are still no approved vaccines for ZIKV (46, 47).

Preclinical studies with saRNA vaccines against various pathogens, including influenza, SARS-CoV-2, HIV, and ZIKV, have yielded promising results (6, 48, 49). These studies demonstrated that saRNA vaccines can induce robust immune responses and reduce viral loads in mouse models of infection (7, 8, 50, 51). The clinical development of vaccines using the saRNA platform is progressing rapidly, and a SARS-CoV-2 saRNA vaccine is currently in phase IIa trials (52). Thus, although less mature than conventional mRNA vaccine technology, the saRNA vaccine platform holds great promise for the future. Ideally, independent strategies would also be developed in parallel to boost T cell immunity or enhance the durability of protection conferred by saRNA vaccines; notably, this topic is only just beginning to be explored.

In the present study, we generated an saRNA vaccine encoding the mature membrane (M) and envelope (E) of ZIKV and assessed its ability to reduce viral burden and to elicit short- and long-term humoral and cellular immune responses in immunocompetent (C57BL/6 wild-type, WT) and immunocompromised (C57BL/6 *Ifnar1*^−/−^) mice. We further explored the effects of Ab-mediated agonism of OX40 and 4-1BB on the duration of vaccine-induced immunity. Our results demonstrate that single-dose immunization with ZIKV M/E vaccine followed by OX40 and 4-1BB agonist Ab administration on consecutive days markedly boosts the production of ZIKV-specific polyfunctional and cytotoxic CD8^+^ T cell responses and prolongs the ability of these T cells to reduce viral loads following infection. These findings provide insights into strategies for improving saRNA vaccine efficacy and durability, with broader implications for vaccine development against not only ZIKV but also other emerging/reemerging pathogens.

## Results

### An saRNA vaccine encoding ZIKV M/E antigen reduces viral loads in ZIKV-challenged mice.

**Figure 1:**
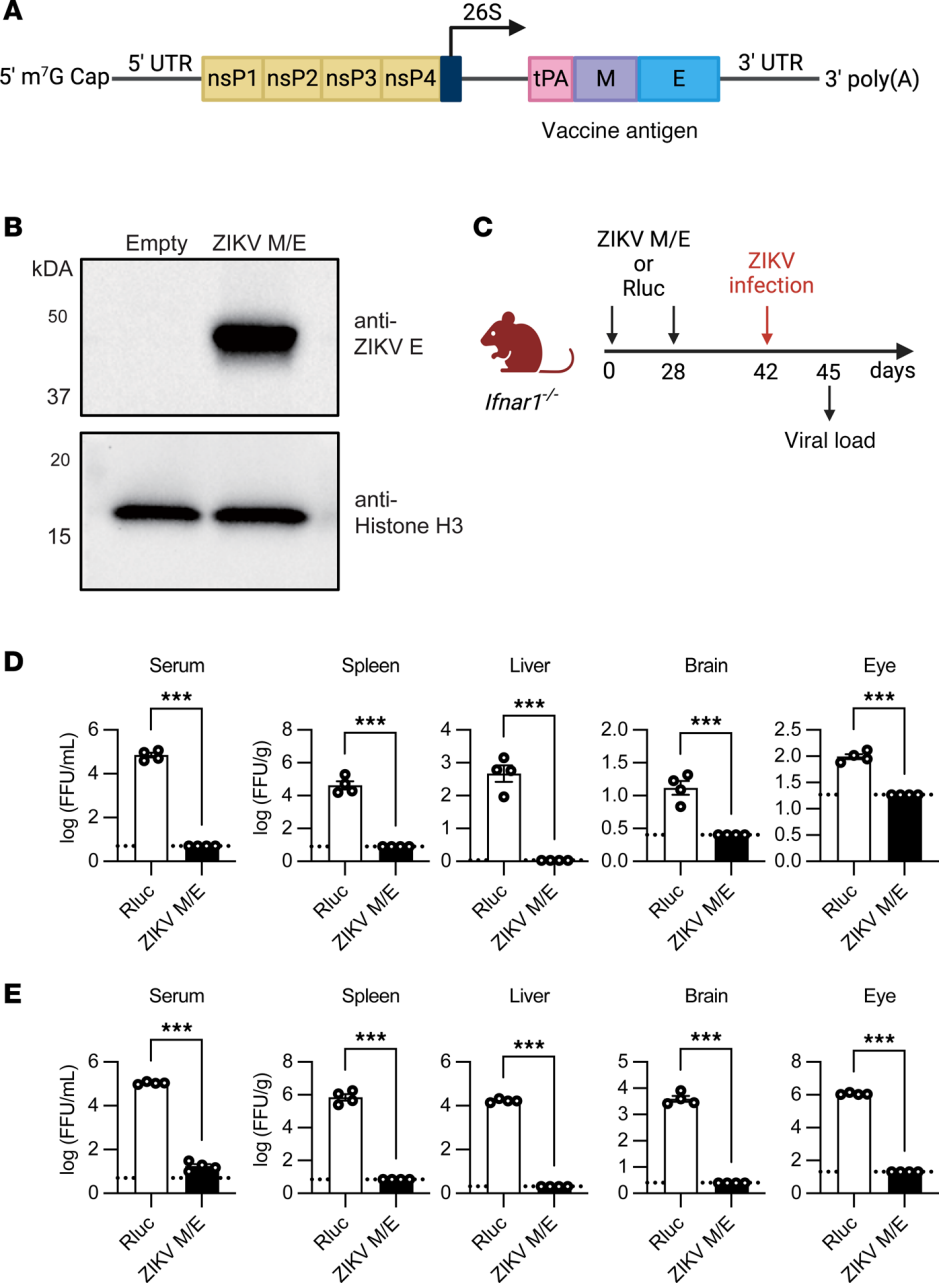
ZIKV M/E saRNA vaccine design and protective efficacy. (**A**) Schematic of the ZIKV M/E saRNA vaccine. The structural genes of Venezuelan equine encephalitis virus (VEEV; strain TC-83) were replaced with the M (without precursor) and E genes from ZIKV SPH2015 downstream of the viral 26S promoter. The 4 VEEV nonstructural proteins (nsP1–4), which encode the replicase, were retained, and the tissue plasminogen activator (tPA) signal sequence was added to facilitate antigen secretion. (**B**) Western blot analysis of whole-cell lysates of untransfected HEK293T cells (empty) or cells transfected with ZIKV M/E RNA probed with anti-ZIKV E or anti–histone H3 mAbs. Histone H3 served as an internal loading control. (**C**) Experimental protocol: *Ifnar1*^−/−^ C57BL/6 mice were immunized twice (days 0 and 28) with ZIKV M/E or Rluc vaccine (5 μg, intramuscularly), then retro-orbitally challenged on day 42 with ZIKV SD001. Blood and tissues were harvested on day 3 postinfection, and viral load was analyzed by focus-forming assay (FFA). (**D** and **E**) Quantification of ZIKV infectious particles in the indicated tissues after infection with 10^3^ (**D**) or 10^6^ (**E**) focus-forming units (FFU) of ZIKV SD001. Data are presented as the mean ± SEM of *n* = 4 mice/group. Circles represent individual mice. Dotted line indicates the limit of detection. ***P < 0.001 by the unpaired *t* test.

To develop an saRNA vaccine against ZIKV with the potential for improved efficacy and durability, we replaced the structural genes of Venezuelan equine encephalitis virus (VEEV) with sequences encoding the mature ZIKV M/E protein while retaining the 4 nonstructural genes of VEEV (Figure 1A). A control vaccine (*Renilla* luciferase; Rluc) was constructed in a similar manner, except the Rluc-encoding sequence was inserted. The quality of the ZIKV M/E saRNA and its ability to produce ZIKV M/E protein after transfection into HEK293T cells were assessed by RNA electrophoresis (Supplemental Figure 1A; supplemental material available online with this article; https://doi.org/10.1172/jci.insight.187405DS1), Western blotting (Figure 1B), and immunofluorescence staining (Supplemental Figure 1B). Rluc and ZIKV M/E saRNAs were then encapsulated into lipid nanoparticles (LNPs), and encapsulation efficiencies of more than 90% for both the Rluc and ZIKV M/E vaccines were confirmed using the RiboGreen assay (Supplemental Figure 1C).

Evaluation of vaccine-induced protection against ZIKV infection and induction of an immune response would ideally be performed using immunocompetent WT mice; however, ZIKV does not replicate well in WT mice because the virus is unable to antagonize the murine type I IFN antiviral response, as it does in humans (53). Therefore, we examined protection against ZIKV infection in the more stringent *Ifnar1*^−/−^ (C57BL/6) mouse strain, which lacks IFNRs and thus supports robust ZIKV replication (54). Vaccine efficacy was assessed based on the levels of infectious virus and viral RNA, as measured by focus-forming assay (FFA) and quantitative real-time reverse transcription PCR (qRT-PCR), respectively. Groups of 6- to 8-week-old male and female (~1:1 ratio) *Ifnar1*^−/−^ mice were immunized intramuscularly (i.m.) with 5 μg ZIKV M/E or Rluc control vaccine on days 0 and 28 and challenged retro-orbitally with 10^3^ or 10^6^ focus-forming units (FFU) of ZIKV SD001 on day 42. Tissue samples were then collected at 3 days postinfection (dpi) and analyzed for infectious virus titers using a cell-based FFA and for viral RNA using qRT-PCR (Figure 1C). Mice primed and boosted with ZIKV M/E vaccine and challenged with ZIKV at a low dose (10^3^ FFU; Figure 1D and Supplemental Figure 1D) or a high dose (10^6^ FFU; Figure 1E and Supplemental Figure 1E) had strikingly reduced viremia and tissue viral loads compared with mice that were immunized with Rluc control vaccine. Indeed, infectious particles were undetectable or virtually undetectable after low- or high-dose challenge, respectively, in mice vaccinated with ZIKV M/E compared with Rluc (Figure 1, D and E). A similar pattern was observed when viral RNA was quantified by qRT-PCR. Compared with control mice, ZIKV M/E–vaccinated mice exhibited significantly reduced RNA levels in sera but undetectable levels in the organs of mice challenged with a low dose of ZIKV (Supplemental Figure 1D) and significantly reduced but detectable RNA levels in the sera and organs of mice challenged with a high dose of ZIKV (Supplemental Figure 1E). Taken together, these results indicate that ZIKV M/E saRNA vaccine can substantially reduce viral burden in this highly susceptible mouse strain.

### ZIKV M/E saRNA vaccine elicits antigen-specific humoral and cellular immunity in mice.

Although a large body of work on immunity to flaviviruses has shown that WT C57BL/6 and *Ifnar1*^−/−^ C57BL/6 mice develop quantitatively and qualitatively similar immune responses, at least as measured in blood and lymphoid organs (55, 56), it is formally possible that the immune response to ZIKV M/E vaccine may be skewed by the absence of IFNRs in *Ifnar1*^−/−^ mice. Therefore, we evaluated the immunogenicity of ZIKV M/E vaccine in immunocompetent WT C57BL/6 mice.

**Figure 2:**
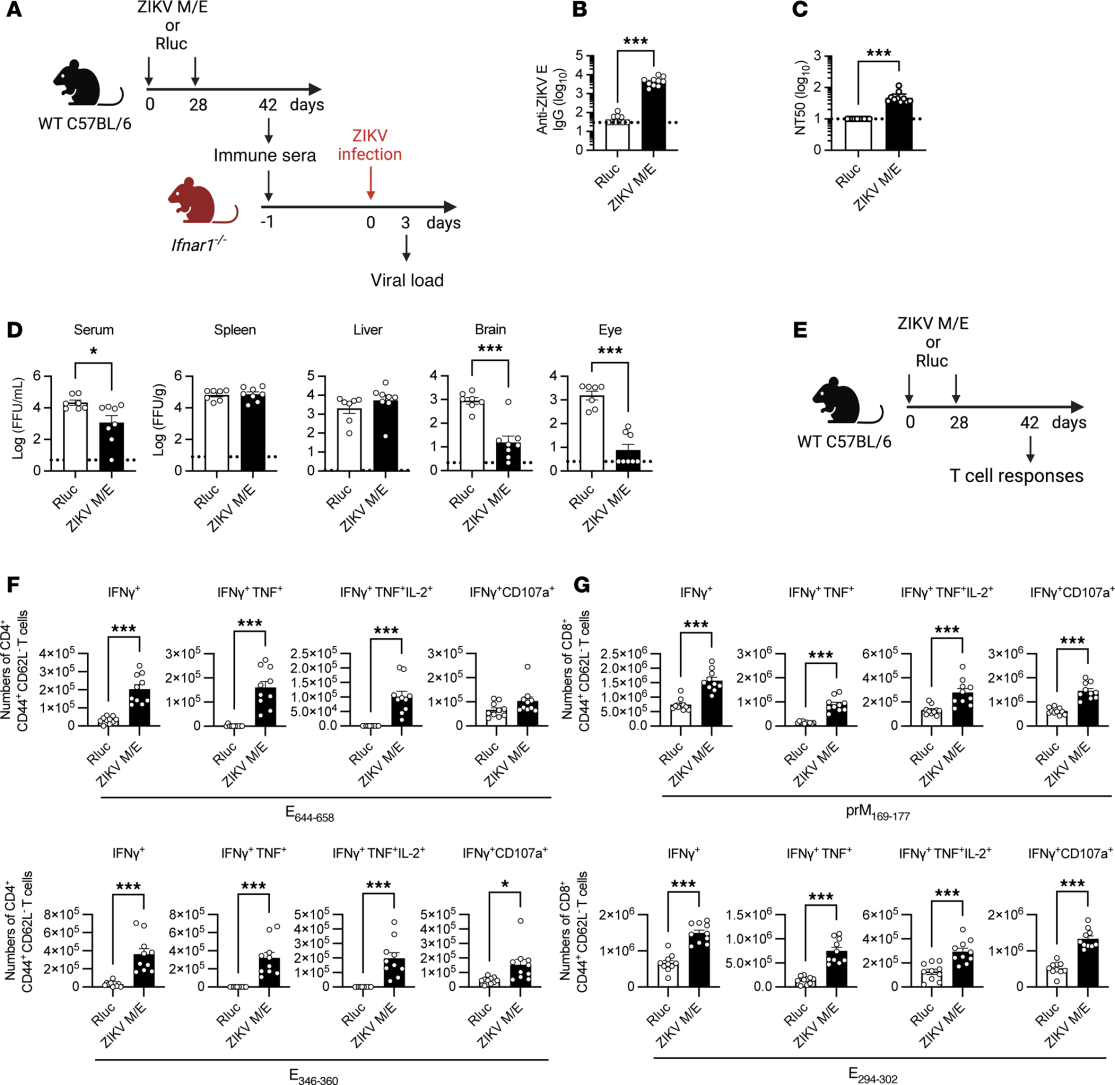
Immunogenicity of ZIKV M/E saRNA vaccine. (**A**) Experimental protocol: Wild-type (WT) mice were immunized twice (days 0 and 28) with ZIKV M/E or Rluc vaccine (5 μg, intramuscularly) and bled on day 42. Sera were prepared, pooled, and injected intraperitoneally (400 μL/mouse) into naive *Ifnar1*^−/−^ mice, which were retro-orbitally challenged 1 day later with 10^3^ FFU of ZIKV SD001. Blood and organs were harvested on day 3 postinfection, and viral loads were analyzed by FFA. (**B** and **C**) Quantification of anti-ZIKV E IgG titers (**B**) and neutralizing antibody titers (**C**). (**D**) Quantification of ZIKV infectious particles in the indicated tissues. (**E**) Experimental protocol: WT mice were immunized twice (days 0 and 28) as above, and spleens were harvested on day 42. Splenocytes were stimulated in vitro with the indicated ZIKV peptides and then stained and analyzed by flow cytometry. (**F** and **G**) Number of IFN-γ^+^–producing, polyfunctional (IFN-γ^+^TNF-α^+^ or IFN-γ^+^TNF-α^+^IL-2^+^), and cytotoxic (IFN-γ^+^CD107a^+^) CD4^+^ (**F**) and CD8^+^ (**G**) effector memory T cells (CD3^+^CD4^+^CD44^+^CD62L^−^) (see Supplemental Figure 7 for gating strategy). Data are pooled from 2 independent experiments and are presented as the mean ± SEM. In panel **D**, for *Ifnar1*^−/−^ mice, *n* = 7 for the Rluc group, and *n* = 8 for the ZIKV M/E group. In panels **B**, **C**, **F**, and **G**, for WT mice, *n* = 10 per group. Circles represent individual mice. Dotted line indicates the limit of detection. ***P < 0.001 by the Mann-Whitney test (**B** and **C**). *P < 0.05, ***P < 0.001 by the unpaired *t* test (**D**, **F**, and **G**).

We first assessed ZIKV-specific Ab responses following prime-boost vaccination (Figure 2A). The ZIKV M/E vaccine induced robust humoral immunity, as evidenced by high titers of both anti-ZIKV E IgG (Figure 2B) and serum neutralizing Abs (nAbs; Figure 2C). Notably, however, recent work has demonstrated a lack of concordance between anti-flaviviral nAb titers measured by in vitro assays and the ability of nAbs to protect against flavivirus infection in vivo (57). Therefore, we also evaluated the capacity of vaccine-elicited Abs to decrease viral loads in passive transfer experiments by injecting immune sera into naive *Ifnar1*^−/−^ mice and infecting the mice with ZIKV 1 day later (Figure 2A). Measurement of infectious ZIKV levels at 3 dpi showed reduced viremia and lower viral burden in the brain and eye, but not in the spleen and liver, of *Ifnar1*^−/−^ mice that received sera from ZIKV M/E–vaccinated compared with Rluc control–vaccinated WT mice (Figure 2D and Supplemental Figure 2A). Thus, ZIKV M/E vaccine effectively elicited Abs that reduced ZIKV infection in vivo.

Next, we examined T cell responses in WT mice on day 42 after vaccination with ZIKV M/E or Rluc vaccine (Figure 2E). Splenocytes were prepared and stimulated in vitro for 5 hours with ZIKV E or prM peptides that had previously been shown to be the immunodominant ZIKV epitopes for CD4^+^ or CD8^+^ T cells in C57BL/6 (H-2^b^) mice (58). One peptide each from ZIKV E and prM was used to stimulate CD8^+^ T cells; however, 2 peptides from ZIKV E were used for CD4^+^ T cells because no ZIKV prM epitopes immunostimulatory for CD4^+^ T cells have yet been identified. After incubation, the splenocytes were analyzed by flow cytometry to quantify functional subsets of effector memory CD4^+^ and CD8^+^ T (Tem; CD44^+^CD62L^−^) cells (Supplemental Figure 7A). Priming and boosting with ZIKV M/E vaccine resulted in a significant increase in the abundance of ZIKV-specific CD4^+^ (Figure 2F and Supplemental Figure 2B) and CD8^+^ (Figure 2G and Supplemental Figure 2C) polyfunctional (IFN-γ^+^TNF-α^+^ and IFN-γ^+^TNF-α^+^IL-2^+^) and cytotoxic (IFN-γ^+^CD107^+^) Tem cells compared with the numbers of cells in Rluc control–vaccinated mice. These data indicate that the ability of ZIKV M/E vaccine to reduce tissue viral burden is likely to be mediated by both humoral and cellular immunity.

### Single-dose immunization with ZIKV M/E saRNA vaccine is sufficient to confer short-term, but not long-term, reduction in viral load following ZIKV challenge.

**Figure 3:**
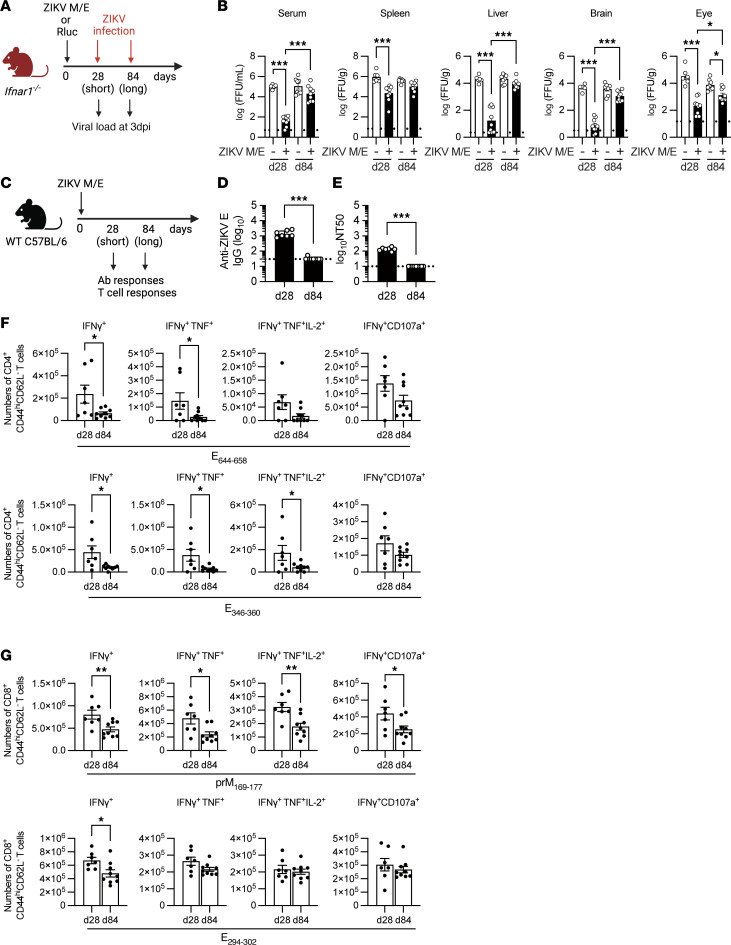
Short- and long-term immunogenicity and protective efficacy of ZIKV M/E saRNA vaccine. (**A**) Experimental protocol: *Ifnar1*^−/−^ mice were immunized once (day 0) with ZIKV M/E or Rluc vaccine (5 μg, intramuscularly) and then retro-orbitally challenged with 10^6^ FFU of ZIKV SD001 on day 28 or 84. Blood and organs were harvested at day 3 postinfection, and viral loads were analyzed by FFA of infectious particles (**B**) in the indicated tissues. (**C**) Experimental protocol: WT mice were immunized once on day 0 as described above, and blood and spleens were collected on either day 28 or day 84. Splenocytes were stimulated in vitro with the indicated ZIKV peptides and then stained and analyzed by flow cytometry. (**D** and **E**) Quantification of anti-ZIKV E IgG titers (**D**) and neutralizing antibody titers (**E**). (**F** and **G**) Number of IFN-γ^+^–producing, polyfunctional (IFN-γ^+^TNF-α^+^ or IFN-γ^+^TNF-α^+^IL-2^+^), and cytotoxic (IFN-γ^+^CD107a^+^) CD4^+^ (**F**) and CD8^+^ (**G**) effector memory T cells (CD3^+^CD4^+^CD44^+^CD62L^−^) (see Supplemental Figure 7 for gating strategy). Data are pooled from 2 independent experiments and are presented as the mean ± SEM. In **B**, for *Ifnar1*^−/−^ mice, *n* = 6 for the Rluc/d28 group, *n* = 9 for the ZIKV M/E/d28 group, *n* = 8 for the Rluc/d84 group, and *n* = 10 for the ZIKV M/E/d84 group. In **D**–**G**, for WT mice, *n* = 7 for the d28 group and *n* = 9 for the d84 group. Circles represent individual mice. Dotted line indicates the limit of detection. *P < 0.05, ***P < 0.001 by 1-way ANOVA with the Holm-Šídák multiple-comparison test (**B**). ***P < 0.001 by the Mann-Whitney test (**D** and **E**). *P < 0.05, **P < 0.01 by the unpaired *t* test (**F** and **G**).

Given the self-replicating nature of the ZIKV saRNA vaccine and the strong immune response observed 2 weeks after priming and boosting, we hypothesized that a single dose of vaccine might be sufficient to elicit protective immunity. To test this, we immunized *Ifnar1*^−/−^ mice on day 0 with ZIKV M/E or Rluc vaccine and then compared short-term (28 days) and long-term (84 days) efficacy against ZIKV challenge (Figure 3A). Compared with the control vaccine, a single dose of ZIKV M/E vaccine significantly reduced ZIKV infectious particles and RNA levels in the sera and organs of mice challenged at 28 days, but not 84 days, after immunization (Figure 3B and Supplemental Figure 3A). These results indicate that immunization with a single dose of ZIKV M/E saRNA vaccine decreases tissue viral burden in the short term, but not the long term, suggesting that immunity wanes rapidly after a single immunization.

To understand the lack of durability of protection, we examined the time course of the vaccine-induced immune response in more detail (Figure 3C). Both humoral and cellular responses showed substantial declines over time, such that titers of anti-ZIKV E IgG and nAbs were significantly lower at day 84 compared with day 28 (Figure 3, D and E), and both CD4^+^ and CD8^+^ T cell responses were also markedly reduced by day 84 (Figure 3, F and G, and Supplemental Figure 3, B and C). These observations explain the transience of single-dose vaccine-induced immunity and highlight the need to develop strategies that enhance the durability of response.

### Cotreatment with OX40 and 4-1BB agonist Abs enhances short-term antigen-specific CD8^+^ T cell responses to ZIKV M/E saRNA vaccine.

**Figure 4:**
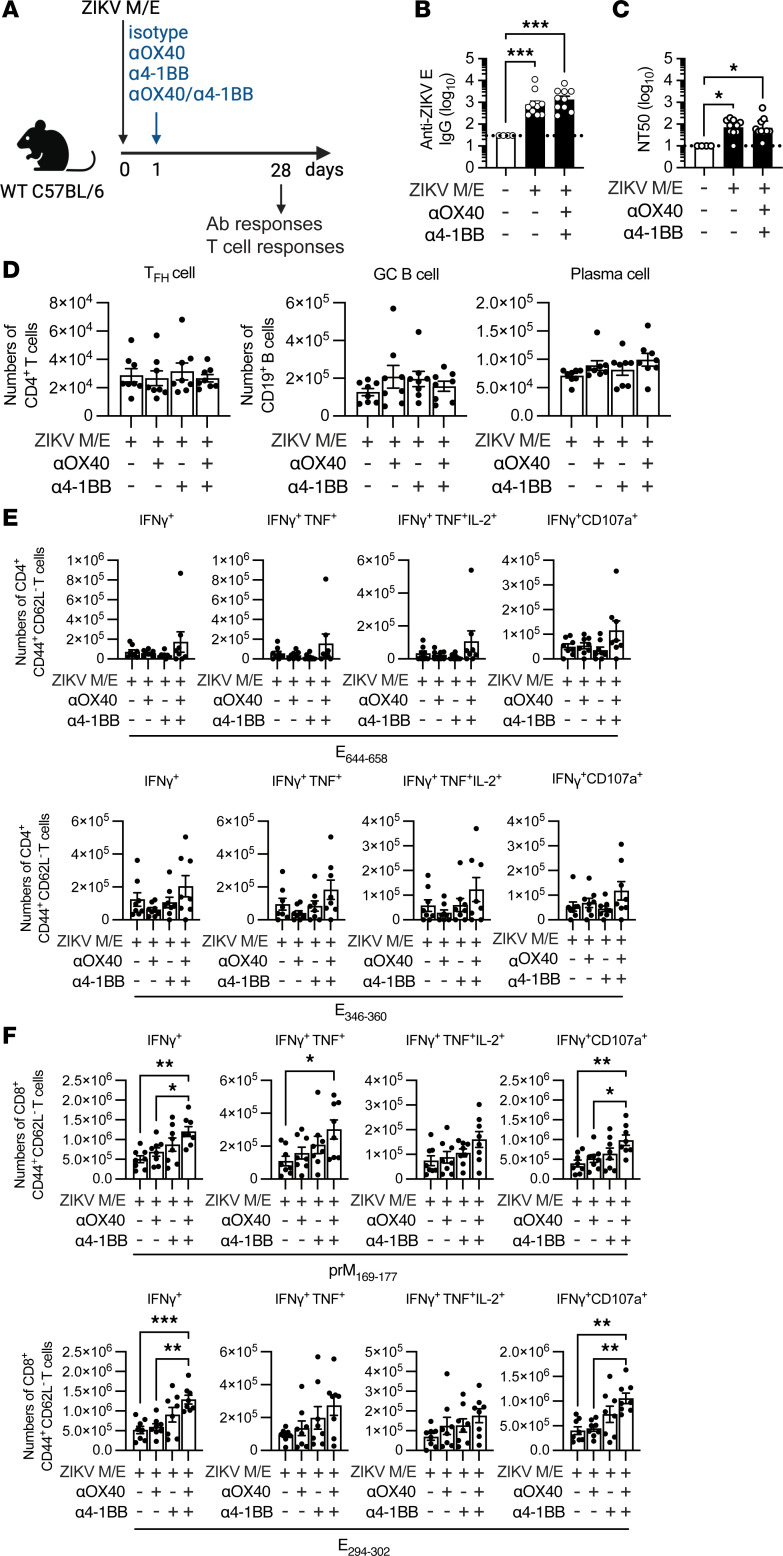
Effect of TNFR agonists on short-term immunogenicity. (**A**) Experimental protocol: Wild-type (WT) mice were immunized once (day 0) with ZIKV M/E vaccine (5 μg, intramuscularly), and 1 day later, mice were injected intraperitoneally with 100 μg anti-(α)OX40, 25 μg α4-1BB, or both αOX40/α4-1BB antibodies or with the same amounts of rat IgG1 and IgG2a isotype control antibodies. Blood and spleens were collected on day 28. Splenocytes were either (**D**) stained and analyzed by flow cytometry or (**E** and **F**) stimulated in vitro with the indicated ZIKV peptides before staining and analysis by flow cytometry (see Supplemental Figure 7). (**B** and **C**) Quantification of anti-ZIKV E IgG titers (**B**) and neutralizing antibody titers (**C**). (**D**) Number of Tfh cells (CD3^+^CD4^+^CXCR5^+^PD-1^+^), germinal center (GC) B cells, (CD19^+^Fas^+^GL7^+^CD138^−^IgD^−^), and plasma cells (CD19^+^CD138^+^IgD^−^). (**E** and **F**) Number of IFN-γ^+^–producing, polyfunctional (IFN-γ^+^TNF-α^+^ or IFN-γ^+^TNF-α^+^IL-2^+^), and cytotoxic (IFN-γ^+^CD107a^+^) CD4^+^ (**E**) and CD8^+^ (**F**) effector memory T cells (CD3^+^CD4^+^CD44^+^CD62L^−^). Data are pooled from 2 independent experiments and are presented as the mean ± SEM of 8 mice/group in total. Circles represent individual mice. Dotted line indicates the limit of detection. *P < 0.05, ***P < 0.001 by the Kruskal-Wallis test (**B** and **C**). *P < 0.05, **P < 0.01, ***P < 0.001 by 1-way ANOVA with the Holm-Šídák multiple-comparison test (**D**–**F**).

As noted in Introduction, stimulation of the TNFR superfamily members OX40 and 4-1BB has been shown to increase Tem cell responses to viral and tumor antigens (29–31), including increased IFN-γ production by both CD4^+^ and CD8^+^ T cells following vaccination of mice against poxvirus (28). To test the possibility that the long-term protective efficacy of our ZIKV M/E vaccine might be similarly boosted by engaging OX40 or 4-1BB, we immunized WT mice with ZIKV M/E vaccine once on day 0 and then injected agonist Abs against OX40 and/or 4-1BB or isotype control Abs intraperitoneally (i.p.) 1 day later (Figure 4A). At 28 days after immunization, we measured the serum Ab response and analyzed splenocytes for Tfh cells, germinal center (GC) B cells, or plasma cells by flow cytometry. Notably, cotreatment with OX40- and/or 4-1BB–specific Abs did not significantly affect titers of anti-ZIKV E IgG or nAbs (Figure 4, B and C) or the numbers or frequencies of Tfh cells, GC B cells, or plasma cells (Figure 4D and Supplemental Figure 4A). Examination of the abundance of CD4^+^ and CD8^+^ Tem cells after in vitro stimulation with ZIKV peptides, as described above, showed no significant changes in the numbers (Figure 4E) or relative frequencies (Supplemental Figure 4B) of polyfunctional or cytotoxic CD4^+^ Tem subsets, though a slightly increased trend was observed in ZIKV M/E–vaccinated mice cotreated with both OX40 and 4-1BB Abs (Figure 4E and Supplemental Figure 4B). In contrast, treatment with anti-OX40 and anti–4-1BB Abs significantly boosted the abundance (Figure 4F) and frequency (Supplemental Figure 4C) of polyfunctional and cytotoxic CD8^+^ Tem cells, with the combination of both Abs resulting in the most robust increases compared with isotype control Ab (Figure 4F and Supplemental Figure 4C). This finding is especially notable, given our previous studies supporting a particularly important role for ZIKV-specific and ZIKV/DENV cross-reactive CD8^+^ T cells in protecting against ZIKV infection (59–63). Therefore, these results suggest that anti-ZIKV CD8^+^ T cell responses elicited by ZIKV M/E saRNA vaccine, and potentially other vaccine platforms, could be significantly improved by coagonism of OX40 and 4-1BB, at least with respect to short-term responses.

### Cotreatment with OX40 and 4-1BB agonist Abs enhances long-term antigen-specific CD8^+^ T cell responses to a ZIKV M/E saRNA vaccine.

**Figure 5:**
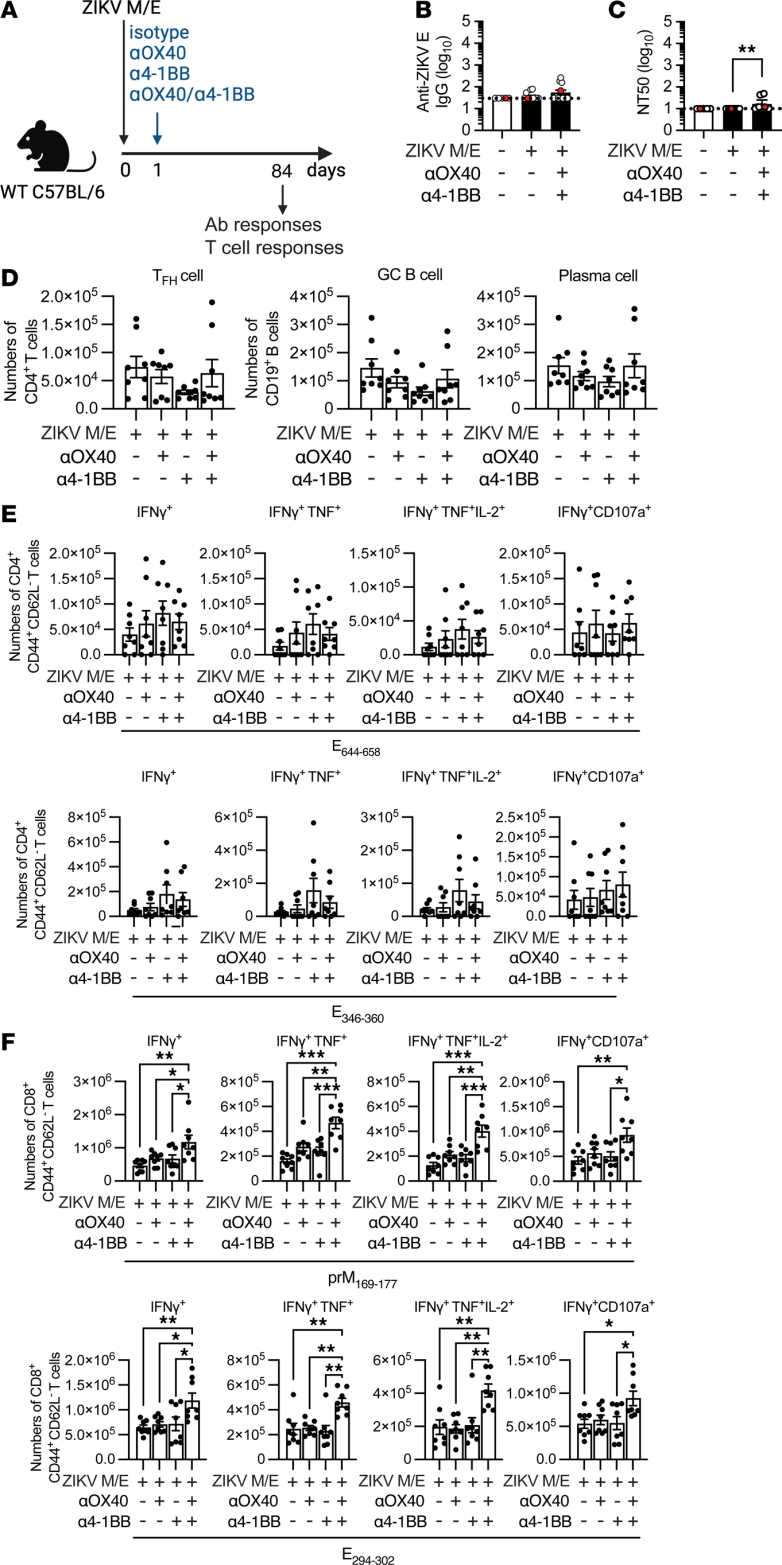
Effect of TNFR agonists on long-term immunogenicity. (**A**) Experimental protocol: Wild-type (WT) mice were immunized once (day 0) with ZIKV M/E vaccine (5 μg, intramuscularly), and 1 day later, mice were injected intraperitoneally with 100 μg αOX40, 25 μg α4-1BB, or both αOX40/α4-1BB antibodies or with the same amounts of rat IgG1 and IgG2a isotype control antibodies. Blood and spleens were collected on day 84. Splenocytes were either (**D**) stained and analyzed by flow cytometry or (**E** and **F**) stimulated in vitro with the indicated ZIKV peptides before staining and analysis by flow cytometry. (**B** and **C**) Quantification of anti-ZIKV E IgG titers (**B**) and neutralizing antibody titers (**C**). The red circles indicate the pooled serum used in Figure 6, E and F. (**D**) Number of Tfh cells (CD3^+^CD4^+^CXCR5^+^PD-1^+^), GC B cells (CD19^+^Fas^+^GL7^+^CD138^−^IgD^−^), and plasma cells (CD19^+^CD138^+^IgD^−^). (**E** and **F**) Number of IFN-γ^+^–producing, polyfunctional (IFN-γ^+^TNF-α^+^ or IFN-γ^+^TNF-α^+^IL-2^+^), or cytotoxic (IFN-γ^+^CD107a^+^) CD4^+^ (**E**) and CD8^+^ (**F**) effector memory T cells (CD3^+^CD4^+^CD44^+^CD62L^−^). Data are pooled from 2 independent experiments and are presented as the mean ± SEM of 8 mice/group in total. Circles represent individual mice. Dotted line indicates the limit of detection. **P < 0.01 by the Kruskal-Wallis test (**B** and **C**). *P < 0.05, **P < 0.01, ***P < 0.001 by 1-way ANOVA with the Holm-Šídák multiple-comparison test (**D**–**F**).

To determine whether ZIKV M/E vaccination combined with OX40/4-1BB agonist Ab treatment can increase the durability of protection, we performed the same experimental protocol as above but evaluated the immune response at day 84 after vaccination (Figure 5A). Similar to the findings on day 28 after vaccination, there were no significant changes in the Ab responses overall (Figure 5, B and C) or the number or frequency of Tfh cells, GC B cells, plasma cells (Figure 5D and Supplemental Figure 5A), or antigen-specific CD4^+^ Tem cells (Figure 5E and Supplemental Figure 5B) in vaccinated mice cotreated with anti-OX40/4-1BB Abs compared with isotype control Ab. However, cotreatment with anti-OX40/4-1BB Abs did induce a robust increase in the abundance and frequency of polyfunctional and cytotoxic ZIKV-specific CD8^+^ Tem cells compared with the control Ab–treated mice and also compared with mice treated with either anti-OX40 or anti–4-1BB alone (Figure 5F and Supplemental Figure 5C). Remarkably, the numbers of cytokine-producing CD8^+^ Tem cells were similar at 28 days and 84 days after immunization (Figure 4F vs. Figure 5F), suggesting that the combined Ab treatment likely extends the durability of vaccine response by maintaining the antigen-specific memory T cell pool. Together, these findings indicate that combinatorial anti-OX40 and anti–4-1BB agonist Ab treatment enhances the durability of antigen-specific CD8^+^ T cell responses to ZIKV M/E saRNA vaccination, presenting a potential strategy for improving vaccine efficacy.

### Cotreatment with OX40 and 4-1BB agonist Abs promotes vaccine durability and long-term efficacy of ZIKV M/E saRNA vaccine in a CD8^+^ T cell–dependent manner.

**Figure 6:**
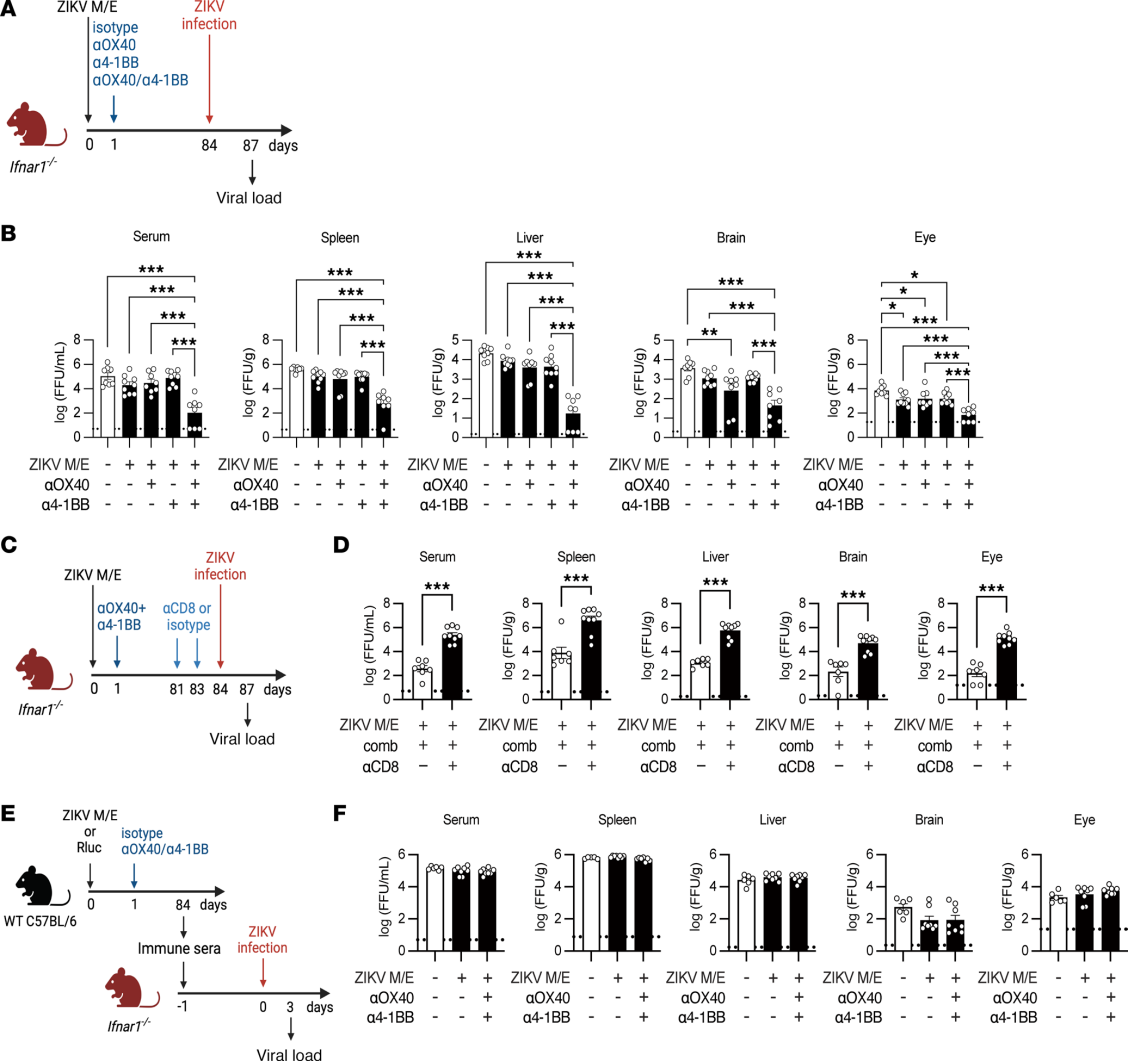
CD8^+^ T cell–driven long-term protection induced by OX40 and 4-1BB cotreatment. (**A**) Experimental protocol: *Ifnar1*^−/−^ mice were immunized (day 0) with the ZIKV M/E vaccine (5 μg, intramuscularly) and treated intraperitoneally 1 day later with 100 μg αOX40, 25 μg α4-1BB, both, or equivalent isotype controls. On day 84, mice were retro-orbitally challenged with 10^6^ FFU of ZIKV. Viral loads were analyzed 3 days later. (**B**) Quantification of ZIKV infectious particles in tissues. (**C**) Experimental protocol: *Ifnar1*^−/−^ mice were immunized, treated, and infected as described in **A**. Prior to the infection, mice were injected intraperitoneally with 300 μg of a CD8-depleting antibody or rat IgG2b isotype control antibody on days 81 and 83. (**D**) Quantification of ZIKV infectious particles in tissues. (**E**) Experimental protocol: Wild-type (WT) mice were immunized and treated as described in **A**. Sera were prepared, pooled, and injected intraperitoneally (400 μL/mouse) into naive *Ifnar1*^−/−^ mice, which were retro-orbitally challenged 1 day later with 10^3^ FFU of ZIKV SD001. Viral loads were analyzed 3 days later. (**F**) Quantification of ZIKV infectious particles in the indicated tissues. Data are pooled from 2 independent experiments and are presented as the mean ± SEM. In **B**, *n* = 8 for the Rluc group, *n* = 9 for the αOX40 or α4-1BB groups, and *n* = 8 for the αOX40/α4-1BB group. In **D**, *n* = 7 for the isotype control group, and *n* = 9 for the αCD8 group. In **F**, *n* = 6 for the Rluc group, and *n* = 8 for the isotype or αOX40/α4-1BB groups. Circles represent individual mice. Dotted line indicates the limit of detection. *P < 0.05, **P < 0.01, ***P < 0.001 by 1-way ANOVA with the Holm-Šídák multiple-comparison test (**B** and **F**). ***P < 0.001 by the unpaired *t* test (**D**).

To determine whether the observed effect of OX40/4-1BB Abs on vaccine-induced CD8^+^ T cell responses at day 84 after immunization translated to improved immunity against viral infection at the same time point, *Ifnar1*^−/−^ mice were immunized once with ZIKV M/E vaccine, treated with OX40/4-1BB agonist Abs 1 day later, and then challenged with ZIKV at 84 days after immunization (Figure 6A). Indeed, we observed a significant reduction in ZIKV burden in sera and organs of mice cotreated with both anti-OX40 and anti–4-1BB Abs compared with control Ab–treated mice but not in mice treated with either agonist Ab alone, though there were some small but significant reductions in the viral burden in the brain and eye of single-Ab–treated mice (Figure 6B and Supplemental Figure 6A). The approximately 2-log reduction in viral load in cotreated mice was of particular interest because our previous work with a related vaccine platform showed that this magnitude of viral reduction in the spleen and serum correlated with only mild clinical manifestations (e.g., ~10% weight loss at 8 days postinfection) and all the mice survived the challenge (58). These findings suggest that the substantial reduction in viral load observed in agonist Ab–cotreated mice would correlate with a mitigated disease course.

To establish the role of CD8^+^ T cells in sustained protection, we performed Ab-mediated depletion of CD8^+^ T cells in *Ifnar1*^−/−^ mice prior to ZIKV challenge at day 84 (Figure 6C). The absence of CD8^+^ T cells, as confirmed by flow cytometry (Supplemental Figure 6B), resulted in significantly increased viral loads in key organs compared with control mice (Figure 6D). However, passive transfer experiments with sera from vaccinated and agonist Ab–treated mice at day 84 after immunization (Figure 6E) failed to decrease viral loads in recipient mice after infection (Figure 6F), indicating that Ab responses did not contribute to anti-ZIKV immunity.

Collectively, these data demonstrate that combinatorial treatment with anti-OX40 and anti–4-1BB agonist Abs promotes long-term immunity against ZIKV infection, primarily through CD8^+^ T cell–dependent mechanisms. Depletion of CD8^+^ T cells significantly compromised anti-ZIKV immunity, while endogenous Ab responses did not contribute to the observed durability. These findings highlight the critical role of T cell responses in mediating viral clearance and provide mechanistic insights into the antiviral immune response elicited by our saRNA vaccine.

## Discussion

The success of mRNA vaccines against COVID-19 has led to the exploration of saRNA vaccines, which have the advantage of amplifying RNA encoding the antigen to enhance expression. Preclinical studies in mice demonstrated that saRNA vaccines induced robust immune responses, even at low doses (8), but they have thus far shown limited efficacy as priming vaccines in human trials, particularly an inability to induce strong Ab responses (64, 65). This discrepancy between rodent and human models underscores the need for further investigation, especially into the cellular immune response elicited by saRNA vaccines. However, saRNA platforms have performed remarkably well as booster vaccines in human trials, surpassing conventional mRNA vaccines in both the magnitude of Ab titer and the durability of response (66). These observations highlight the potential for saRNA technology to enhance and prolong antigen expression, resulting in more robust immune responses. Importantly, further research is required to determine whether these enhanced responses translate into durable protection against challenging infectious diseases, such as those induced by ZIKV and DENV.

Our study demonstrates that the ZIKV M/E saRNA vaccine effectively elicits both humoral and cellular antiviral immunity and reduces ZIKV infection in the short but not long term. Long-term anti-ZIKV immunity was achieved by adding OX40 + 4-1BB agonist Ab treatment to the vaccination protocol, which enhanced polyfunctional and cytotoxic CD8^+^ Tem responses and decreased viral loads at 3 months after vaccination. Thus, an immunization protocol that combines an saRNA vaccine with anti-OX40 + 4-1BB agonism has the potential to address a key challenge in achieving durable immunity compared with current mRNA vaccines (2–4). Boosting vaccine-elicited CD8^+^ T cell responses is particularly relevant for controlling infection by flaviviruses, including ZIKV and DENV, because a robust CD8^+^ T cell response can counter the pathogenic role of subneutralizing anti-flaviviral Abs, which can exacerbate disease through a process known as Ab-dependent enhancement of infection (67–74). Thus, such CD8^+^ T cell responses, both virus specific and cross-reactive, not only contribute to immunity against related flaviviruses and heterotypes but also can do so even in the presence of potential pathogenic Ab responses (63, 75–78).

Although effective in promoting durable antiviral immunity, the vaccination strategy employed in this study consisted of i.m. administration of the vaccine and i.p. administration of the agonist Abs on consecutive days. It is not yet clear whether this temporal separation is essential or whether both the vaccine and Abs could be administered at least at the same site (e.g., i.m. and subcutaneously in the upper arm). Alternatively, mRNA-based adjuvants targeting the OX40 and 4-1BB pathways could be administered with the viral antigen mRNA. This approach would allow administration of a single injection while achieving the same goal of fortifying and sustaining the immune response. Indeed, this concept is supported by a recent study in which IL-12p70 mRNA-LNPs were added to the BNT162b2 SARS-CoV-2 mRNA-LNP vaccine, resulting in enhanced humoral and cellular responses (79). This approach could not only reduce the burden on vaccinees by requiring a single visit but also significantly facilitate vaccine deployment worldwide, particularly in resource-limited settings.

The exact mechanisms by which OX40 and 4-1BB agonist Abs enhanced CD8^+^ T cell responses and contributed to sustained viral load reduction in our study remain to be fully elucidated. Previous work has shown that agonist Abs against either OX40 or 4-1BB can independently enhance antigen-specific CD8^+^ T cell responses to vaccinia virus and poxvirus in mice immunized with viral peptides (19, 21), which contrasts with our finding that the combination of OX40 and 4-1BB agonist Abs had a much greater effect than either Ab alone on CD8^+^ T cell responses, particularly in the long-term experiments. This discrepancy may be due to several factors that differ between the studies, including the specific vaccine platforms, the vaccine and Ab doses, the timing of agonist Ab administration, and the antigen targeted (17, 18, 24, 80).

OX40 and 4-1BB are predominantly expressed on T cells, and their ligands, OX40L and 4-1BBL, respectively, are mainly expressed on antigen-presenting cells (16). Both OX40 and 4-1BB are inducible upon T cell activation; for example, in a study with OT-1 TCR transgenic mice, expression of 4-1BB and, to a lesser extent, OX40 on T cells peaked at 2–4 days after infection with antigen-expressing adenovirus (81). In this regard, a recent study of a SARS-CoV-2 mRNA vaccine efficacy in mice showed that CD8^+^ T cell responses were enhanced only when an anti–4-1BB Ab was administered on day 4 after vaccination, not when the vaccine and Abs were administered at the same time (80). This finding suggests that the precise timing of vaccine and Ab treatment is likely crucial, and future studies should explore the optimal timing to maximize the boosting effects. It is possible that the synergistic effect of OX40/4-1BB agonist Abs may not be mediated by direct agonistic signaling but indirectly via changes in the expression of these TNFR family members. For example, OX40 pathway activation by administration of OX40L-Ig fusion protein was shown to upregulate 4-1BB expression on CD8^+^ T cells induced by SARS-CoV-2 spike protein vaccine in mice, thereby augmenting the immune response (17). Thus, understanding the mechanisms by which signaling through OX40 and 4-1BB, both individually and synergistically, promotes CD8^+^ T cell responses to mRNA vaccines could inform the development of more effective adjuvant strategies for saRNA vaccines.

Another critical question relevant to the mechanism of OX40/4-1BB Ab-mediated enhancement of saRNA vaccine durability is whether the same effect is observed when agonist Abs against other TNFR family members, such as CD40 and GITR, are coadministered with the vaccine. In support of this possibility, coactivation of 4-1BB and CD40 has been shown to induce dendritic cell maturation and enhance CD8^+^ T cell effector function in response to stimulation of human cells with claudin 6 peptides in vitro (82). Similarly, administration of oncolytic adenovirus coexpressing OX40 and CD40 ligands resulted in synergistic activation of OX40 and CD40, induction of tumor-specific CD8^+^ effector T cells, and improved control of melanoma growth in tumor peptide-vaccinated mice (83). Furthermore, combined treatment with OX40 and GITR ligand fusion proteins enhanced both CD4^+^ and CD8^+^ T cell activation and antitumor activity in CT26 colon cancer–bearing mice (84). Thus, the ability to enhance mRNA vaccine–mediated antigen-specific immunity may be a general feature of TNFR members and not limited to OX40 and 4-1BB alone.

In conclusion, our study demonstrates that a ZIKV M/E saRNA vaccine can induce robust antiviral immune responses in mice and that administration of OX40 and 4-1BB agonist Abs can significantly enhance the durability of this response via CD8^+^ T cells. These findings may contribute to the development of more effective mRNA vaccines against ZIKV and other flaviviruses and offer broader insights applicable to other vaccine platforms and infectious diseases.

## Methods

### Sex as a biological variable.

Our study examined male and female animals, and similar results were obtained for both sexes.

### Mice.

WT C57BL/6 mice were purchased from Jackson Laboratory. *Ifnar1*^−/−^ C57BL/6 mice were obtained from Wayne Yokoyama (Washington University in St. Louis, St. Louis, Missouri, USA) and bred under pathogen-free conditions at La Jolla Institute for Immunology.

### mRNA-LNP vaccines.

Vaccines were generated using an alphavirus self-amplifying mRNA vaccine platform derived from VEEV (strain TC-83) (85). Genes encoding the endogenous viral structural proteins were deleted and replaced with RNA encoding ZIKV strain SPH2015 M (precursor sequence removed) and E proteins. For the control vaccine, the endogenous structural genes were replaced with Rluc RNA. DNA constructs containing a T7 promoter site for in vitro transcription were customized and produced by Watsonbio. Briefly, the DNA constructs were digested with NotI-HF (New England Biolabs R3189M), followed by in vitro transcription (Invitrogen AMB13345) to synthesize mRNA. The mRNA was then capped (Thermo Fisher Scientific C-SCCS1710) to increase the translation efficiency and to prevent degradation. mRNA quality was confirmed by electrophoresis (Supplemental Figure 1A). Subsequently, the mRNAs were encapsulated into LNPs (GenVoy-ILM NWW0042 or OZ Biosciences LNP15000) using the NanoAssemblr Benchtop system (Precision NanoSystems). The encapsulation efficiency (Supplemental Figure 1C) and mRNA concentration were determined using a RiboGreen assay (Invitrogen R11490).

### Western blot analysis of ZIKV M/E expression.

HEK293T cells (ATCC, CRL-3216) were transfected with ZIKV M/E RNA (TransIT mRNA transfection kit, Mirus, MIR 2250) or remained untransfected (empty control) for 48 hours, and the cells were then collected, lysed with RIPA buffer, and centrifuged at 16,000*g* for 20 minutes at 4°C. The supernatants were collected, mixed with 2× Laemmli buffer (Bio-Rad, 1610737) supplemented with 5% 2-mercaptoethanol (Gibco, 21985-023), and heated at 95°C for 5 minutes. Samples were separated on 4% to 20% polyacrylamide gels and transferred to a PVDF membrane (MilliporeSigma, IPFL00010). The membrane was blocked with 5% blotting-grade blocker (Bio-Rad, 1706404) and incubated overnight at 4°C with mouse pan-flavivirus E protein-specific mAb 4G2 (Bio X Cell, 1-4G2-4-15). After washing, the membrane was incubated with HRP-conjugated secondary Ab (Jackson ImmunoResearch Laboratories, 115-035-072) for 1 hour at room temperature. Following additional washes, the membrane was incubated with Western Lightning Plus-ECL (PerkinElmer, NEL103001EA), then imaged using a Bio-Rad ChemiDoc system. After imaging, the same membrane was stripped using stripping buffer (Thermo Fisher Scientific, 46430), reblocked, and incubated overnight at 4°C with rabbit anti–histone H3 mAb (Cell Signaling Technology, 4499). The subsequent steps, including secondary Ab (Jackson ImmunoResearch Laboratories, 111-036-003) incubation, detection, and imaging, were repeated.

### Virus preparation and quantification of infectious particles by FFA.

ZIKV SD001, which was isolated in 2016 from a woman who had traveled to Caracas, Venezuela (86), was cultured in C6/36 *Aedes albopictus* mosquito cells (ATCC, CRL-1660) as described previously (75). Viral titers in culture media or mouse sera/tissue samples were measured using a BHK-21 cell–based FFA as previously described (58). Briefly, BHK-21 cells (ATCC, CCL10) were resuspended in supplemented MEMα medium (10% FBS, 1% penicillin-streptomycin, and 1% HEPES), seeded at 2 × 10^5^ cells/well in 24-well plates, and incubated overnight at 37°C in a humidified 5% CO_2_ atmosphere. The cells were then infected with serial dilutions of virus-containing culture media, sera, or tissue homogenates (prepared using a QIAGEN TissueLyser II) for 2 hours at 37°C with gentle rocking. The supernatants were removed, the cells were overlaid with fresh medium containing 1% carboxymethyl cellulose (MilliporeSigma, C9481), and the plates were incubated for an additional 3 days. The cells were then fixed with 4% formalin (Thermo Fisher Scientific, F79-1), permeabilized with 1% Triton X-100 (MilliporeSigma, T8787), blocked with 10% FBS in PBS, and incubated with pan-flavivirus E protein-specific monoclonal Ab 4G2 (Bio X Cell, D1-4G2-4-15) for 1 hour followed by HRP-conjugated goat anti-mouse IgG (Jackson ImmunoResearch Laboratories, 115-035-072) for 1.5 hours. Viral foci were visualized using True Blue substrate (KPL, SeraCare, 5510-0030) and manually counted. Data are expressed as FFU/g tissue or FFU/mL serum. The limit of detection, as indicated in the figures, was defined as the minimum number of foci detected normalized to the average tissue weight. Negative data points were assigned a value equal to the limit of detection.

### Mouse vaccination and infection.

Groups of age-matched (6–8 weeks) and sex-matched (~1:1 ratio) C57BL/6 WT or *Ifnar1*^−/−^ mice were immunized i.m. in the quadriceps muscles of both hind limbs with 5 μg of Rluc or ZIKV M/E vaccine on day 0 alone or on days 0 and 28, as indicated in the figure legends. For protective efficacy experiments, *Ifnar1*^−/−^ mice were immunized on day 0 alone or days 0 and 28, then infected retro-orbitally with 10^3^ or 10^6^ (the highest dose that can be administered in the injection volume) FFU of ZIKV SD001 (86) on day 28, 42, or 84 as indicated. Mice were euthanized by CO_2_ inhalation at 3 dpi, blood samples were collected, and the mice were then perfused with PBS. Organs were collected and stored until analysis either in MEMα medium (Gibco, 12-561-072) at −80°C for subsequent FFAs or in RNA*later* (Thermo Fisher Scientific, AM7021) at 4°C for RNA isolation and qRT-PCR. For immune response experiments, WT mice were immunized on days 0 and 28 as described above and euthanized on day 42. The spleens were collected in 10% FBS/RPMI medium, kept at 4°C, and immediately processed by pressing through cell strainers (Thermo Fisher Scientific, 22-363-548) for splenocyte isolation. For the anti-OX40 and anti–4-1BB Ab experiments, WT or *Ifnar1*^−/−^ mice were immunized on day 0 as described above; injected i.p. on day 1 with either control Abs, 100 μg of IgG1 (Bio X Cell, BE0088) and 25 μg of IgG2a (Bio X Cell, BE0089), or with 100 μg anti-OX40 (Bio X Cell, BE0031), and/or 25 μg anti-4-1BB (Bio X Cell, BE0239); and then analyzed for immune responses on day 28 or 84 (WT mice) or infected with ZIKV on day 84 and analyzed for tissue viral load on day 87 (*Ifnar1*^−/−^ mice), as described above.

### ELISA.

High-binding affinity ELISA plates (96-well; Corning, 9018) were coated with ZIKV E protein (2 mg/mL, Native Antigen, ZIKVSU-ENV) in coating buffer (0.1 MNaHCO_3_) overnight at 4°C and then blocked for 1 hour at room temperature with 5% Blocker Casein in PBS (Thermo Fisher Scientific, 37528). Mouse serum samples were diluted 3-fold (from 1:30 to 1:7,290) in 1% BSA/PBS, added to the coated wells, and incubated for 1.5 hours at room temperature. Plates were then washed 3 times with wash buffer (0.05% Tween 20 [Promega, H5151] in PBS), and HRP-conjugated goat anti-mouse IgG Fc (1:5,000 in 1% BSA/PBS, Jackson ImmunoResearch Laboratories, 209-005-088) was added to each well for 1.5 hours at room temperature. TMB chromogen solution (Thermo Fisher Scientific, N301) was added to the wells, the reaction was stopped by addition of 2N sulfuric acid, and the absorbance at 450 nm was read immediately on a SpectraMax M2E microplate reader (Molecular Devices). ZIKV-specific endpoint titers were calculated as the reciprocal of the highest serum dilution that gave a reading twice the cutoff absorbance of the negative control (1% BSA/PBS).

### nAb assay.

Serum samples were heat-inactivated for 30 minutes at 56°C and then diluted 5-fold (from 1:10 to 1:31,250) in serum-free RPMI medium supplemented with 1% penicillin-streptomycin and 1% HEPES buffer, in 96-well, round-bottom plates. Mature ZIKV SD001 was prepared by infecting human Vero cells that overexpress human furin (87), which cleaves the pr peptide and enhances the maturation efficiency of infectious ZIKV. A pretitrated aliquot of mature ZIKV SD001 (optimized to induce infection in 7%–15% of cells) was mixed with the diluted serum samples and incubated for 1 hour at 37°C. U937-DC-SIGN cells (ATCC, CRL-3253) were then added to the serum/virus mixture at 10^5^ cells per well and incubated for 2 hours at 37°C. Positive control cells were incubated with virus in the absence of serum, and negative control cells were incubated without virus. Following the incubation, the plates were centrifuged at 300*g*, the supernatants were aspirated, and fresh complete RPMI medium (supplemented with 10% FBS, 1% penicillin-streptomycin, and 1% HEPES buffer) was added. The plates were incubated for an additional 20 hours at 37°C, after which the cells were harvested and stained with PE-conjugated anti-CD209 (BD Biosciences, 551265). Cells were then fixed and permeabilized using Cytofix/Cytoperm solution (BD Biosciences, 554722), followed by intracellular staining with Alexa Fluor 647–conjugated 4G2 mAb (in-house conjugated). Data were acquired using a FACSCanto II flow cytometer (BD Biosciences) and analyzed with FlowJo software X 10.10.0 (TreeStar). Neutralization percentage was calculated as previously described (58), and data are presented as the log serum dilution resulting in 50% neutralization.

### Serum passive transfer.

WT mice were vaccinated twice on days 0 and 28, as described in Figure 2A, and blood samples were collected on day 42. Alternatively, WT mice were vaccinated and treated with agonist antibodies, as described in Figure 6E, and blood samples were collected on day 84. Sera from each group were pooled and injected i.p. (400 μL/mouse) into *Ifnar1*^−/−^ mice. The next day, the mice were infected retro-orbitally with 10³ FFU of ZIKV SD001, and blood and organs were harvested for viral load quantification at 3 dpi.

### Quantification of viral RNA.

RNA was isolated from mouse sera using the QIAmp Viral RNA Mini Kit (QIAGEN, 52906) and from tissues using the RNeasy Mini Kit (QIAGEN, 74106). qRT-PCR was performed using the qScript One-Step qRT-PCR Kit (Quanta Bioscience, 76047-080) with a CFX96 Touch real-time PCR detection system (Bio-Rad, CFX Manager 3.1). Amplification was performed with ZIKV-specific primers, as described previously (88), with the following cycling conditions: 45°C for 15 minutes, 95°C for 15 minutes, followed by 40 cycles of 95°C for 15 seconds and 60°C for 15 seconds, and a final extension of 72°C for 30 minutes. Viral RNA concentrations were determined using a standard curve generated from four 100-fold serial dilutions of in vitro–transcribed RNA from ZIKV strain FSS13025. Data are expressed as viral RNA levels normalized to 18S rRNA levels in the same samples. The limit of detection, as indicated on the figures, was determined by the copy number of the water control sample and normalized to the average 18S rRNA levels. Negative data were assigned a value equal to the limit of detection.

### Intracellular cytokine staining.

Tem cells (viable CD3^+^CD4^+^/CD8^+^CD44^+^CD62L^−^), Tfh cells (viable CD3^+^CD4^+^CD44^+^CD62L^−^CXCR5^+^PD-1^+^), GC B cells (viable CD19^+^Fas^+^GL7^+^CD138^−^IgD^−^) cells, and plasma cells (viable CD19^+^CD138^+^IgD^−^) were identified and quantified by flow cytometry. The full gating strategy is shown in Supplemental Figure 7.

For quantification of Tem cells, splenocytes were resuspended in 10% FBS/RPMI medium, plated at 2 × 10^6^ cells/well in 96-well plates, and incubated for a total of 5 hours with 2 μg/well of ZIKV E or M peptides as previously described (58). After 1-hour incubation, brefeldin A (BioLegend, 420601) and anti-CD107a PE (clone eBio1D4B, eBioscience, 12-1071-83) were added to the cells, and the incubation was continued for the remaining 4 hours. All experiments included positive controls incubated with PMA and ionomycin (eBioscience, 00-4970-93) and negative controls incubated with medium alone. After the incubation, peptide-stimulated or control cells were stained with viability dye (eBioscience, 65-0868-14), blocked with FcBlocker (CD16/CD32 mAb 2.4G2; BD, 553142), and stained with the following fluorophore-conjugated mAbs: anti-CD3e PE-Cy7 (clone 145-2C11, eBioscience, 25-0031-82), anti-CD4 BUV395 (clone GK1.5, BD Bioscience, 565974), anti-CD8a BV510 (clone 53-6.7, BioLegend, 100751), anti-CD44 BV785 (clone IM7, BioLegend, 103041), and anti-CD62L APC eFluor 780 (clone MEL-14, eBioscience, 47-0621-82). The cells were then fixed and permeabilized with Cytofix/Cytoperm (BD Biosciences, 554722) and stained intracellularly with anti–IFN-γ FITC (clone XMG1.2, Tonbo Biosciences, 35-7311-U100), anti–TNF-α APC (clone MP6-XT22, eBioscience, 17-7321-82), and anti–IL-2 BV711 (clone JES6-5H4, BioLegend, 503837) mAbs.

For quantification of Tfh cells, unstimulated splenocytes were stained with viability dye and blocked with FcBlocker as above, then surface-labeled with anti-CD3e PE-Cy7 (clone 145-2C11, eBioscience, 25-0031-82), anti-CD4 APC eFluor 780 (clone GK1.5, eBioscience, 47-0041-82), anti-CD44 BV785 (clone IM7, BioLegend, 103041), anti-CD62L Alexa Fluor 700 (clone MEL-14, BioLegend, 104426), anti-CXCR5 BV421 (clone L138D7, BioLegend, 145512), and anti–PD-1 BV605 (clone 29F.1A12, BioLegend, 135220). For GC B and plasma cells, unstimulated splenocytes were stained with viability dye and blocked with FcBlocker as above, then surface-labeled with anti-CD19 PE (clone eBio1D3, eBioscience, 12-0193-82), anti-CD138 PerCP Cy5.5 (clone 281-2, BioLegend, 142510), anti-IgD FITC (clone 11-26c.2a, BD Biosciences, 553439), anti-GL7 Alexa Fluor 647 (clone GL7, BD Biosciences, 561529), and anti-Fas CD95 BV510 (clone Jo2, BD Biosciences, 563646). Data were acquired using an LSRFortessa or LSRFortessa X-20 flow cytometer (BD Biosciences) and analyzed using FlowJo software X 10.10.0.

### Depletion of CD8^+^ T cells.

Vaccinated and agonist Ab–treated *Ifnar1*^−/−^ mice were injected i.p. with 300 μg of a CD8-depleting Ab (clone 2.43, Bio X Cell, BE0061) or isotype control Ab (Bio X Cell, BE0090) on days 81 and 83 prior to ZIKV infection, as illustrated in Figure 6C. The efficiency of CD8^+^ T cell depletion was confirmed by flow cytometry (Supplemental Figure 6B), using splenocytes stained with a different anti-CD8 Ab (clone 53-6.7, BioLegend, 100751), to ensure accurate assessment of depletion.

### Statistics.

Data were analyzed using Prism software v10.2.1 (GraphPad Software) and are presented as the mean ± SEM. All data, except for the Ab titer data, were found to be normally distributed based on the Kolmogorov-Smirnov and Shapiro-Wilk tests. Group means were compared with either unpaired 2-tailed *t* test (for 2 groups) or 1-way ANOVA with the Holm-Šídák multiple-comparison test (for 3 or more groups). Antibody titers were analyzed using the Mann-Whitney *U* test (for 2 groups) or the Kruskal-Wallis test (for 3 groups). *P* < 0.05 was considered to indicate statistical significance.

### Study approval.

Mouse studies were performed at La Jolla Institute for Immunology following biosafety level 2 guidelines with approval of the Institutional Animal Care and Use Committee under protocol AP00001029. All experiments were performed in strict accordance with recommendations set forth in the NIH *Guide for the Care and Use of Laboratory Animals* (National Academies Press, 2011).

### Data availability.

All data generated or analyzed during this study are included in the main article and supplement. Additionally, supporting data values — including individual data points for all graphs and values underlying reported means — are provided in a separate Excel (XLS) file with distinct tabs corresponding to each figure panel. This file, titled “Supporting Data Values,” is available as part of the supplement.

## Author contributions

HHL, MC, and SS designed the study. HHL designed and performed the experiments, analyzed the data, and wrote the manuscript. RPDSA, QHL, LE, JT, HM, KC, KVV, A Singh, LLT, and A Street performed the experiments. HHL, QHL, AEN, MC, and SS interpreted the data and edited the manuscript. MC and SS supervised the study.

## Supplementary Material

Supplemental data

Unedited blot and gel images

Supporting data values

## Version 1 04/03/2025

In-Press Preview

## Version 2 05/22/2025

Electronic publication
