# Supplementary material for: Enhanced durability of a Zika virus self-amplifying RNA vaccine through combinatorial OX40 and 4-1BB agonism
Source: JCI Insight. 2025 Apr 3;10(10):e187405. doi: 10.1172/jci.insight.187405 (PMC12128976; doi:10.1172/jci.insight.187405)
Supplement: Supplemental data [file jciinsight-10-187405-s068.pdf]

Supplementary Figures

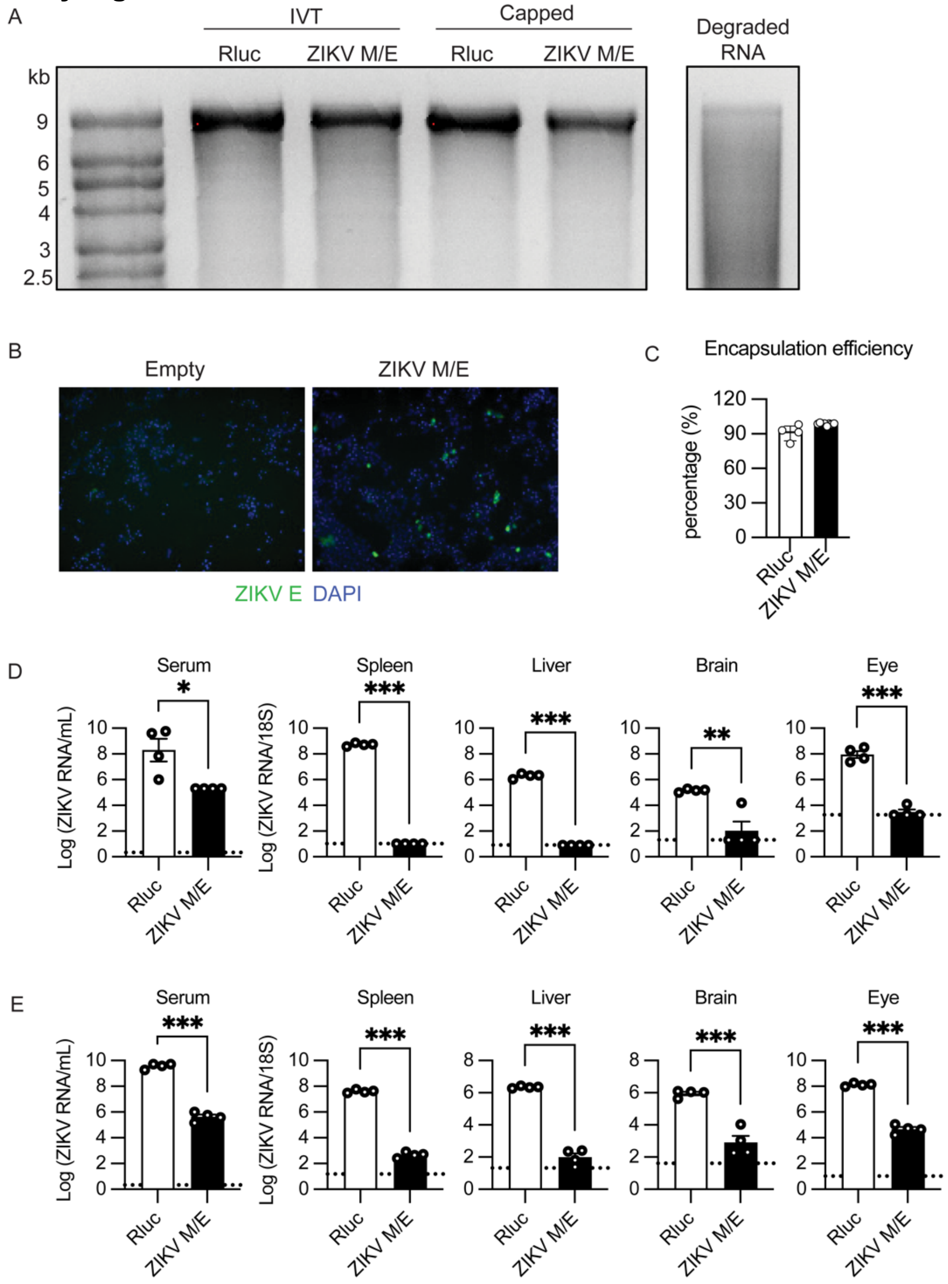

Fig S1. ZIKV M/E saRNA vaccine validation and evaluation of protective efficacy.

(A) RNA gel electrophoresis of saRNA for vaccine preparation. Lane 1, size markers; lanes 2 and 3, in vitro transcription products of Rluc (lane 2) and ZIKV M/E (lane 3) RNA; lanes 4 and 5, capped products of Rluc (lane 4) and ZIKV M/E (lane 5) RNA. Right-hand panel shows an example of degraded RNA. (B) Immunofluorescence images of untransfected 293T cells

(empty) or cells transfected with ZIKV M/E RNA and probed with anti-ZIKV E antibody (green). Nuclei were stained with DAPI (blue). (C) Encapsulation efficiency of Rluc control and ZIKV M/E vaccines. (D and E) Data were collected from the same animal experiment shown in Figure 1C. qRT-PCR analysis of ZIKV RNA levels in the indicated organs of *Ifnar1*$^{-/-}$ mice on day 3 after retro-orbital challenge with 10^3 (D) or 10^6 (E) focus-forming units of ZIKV SD001. Data are presented as the mean ± SEM of 4 mice/group. Circles represent individual mice. Dotted line indicates the limit of detection. *$P < 0.05$, **$P < 0.01$, ***$P < 0.001$ by the unpaired t-test.

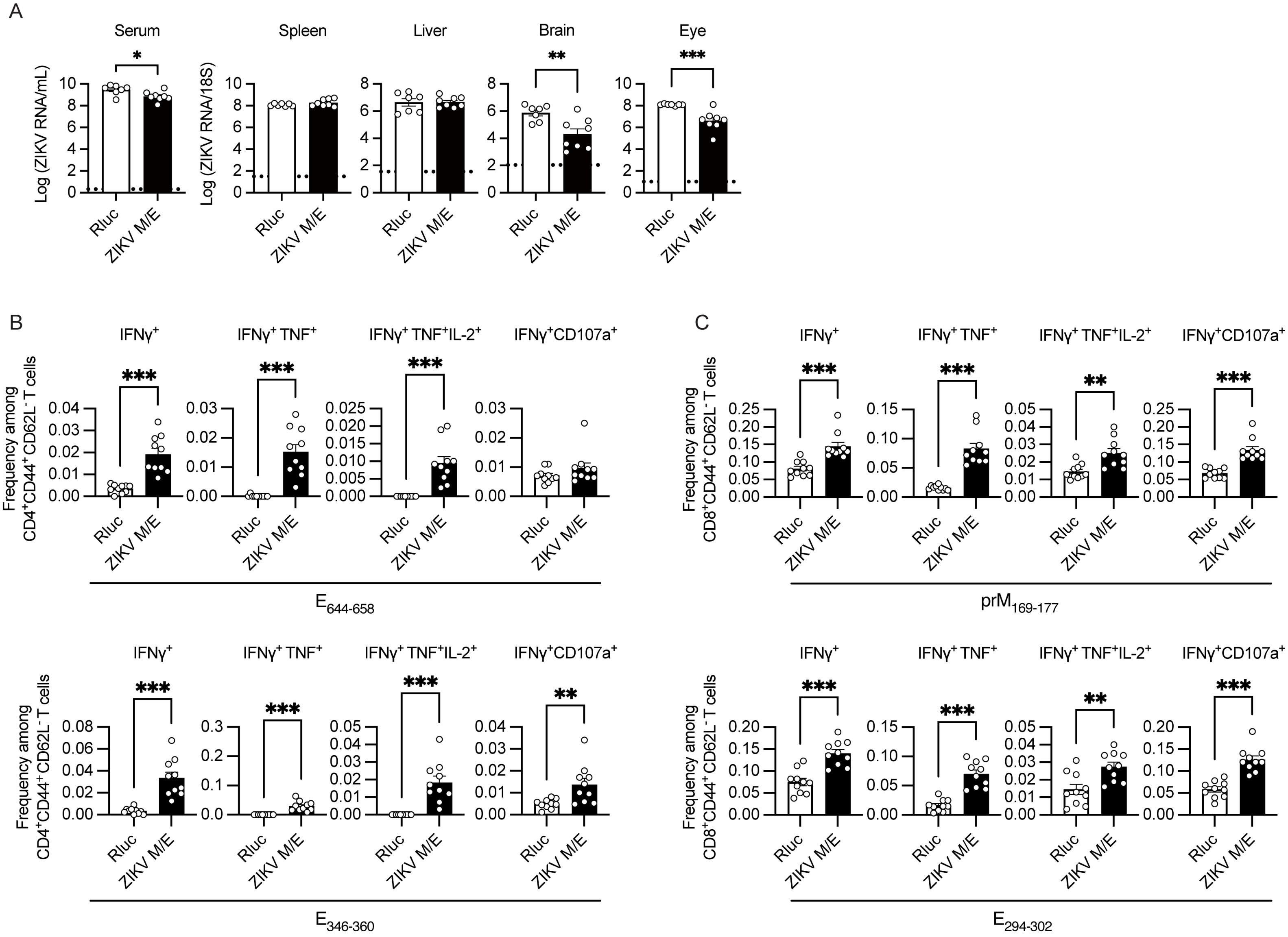

Fig S2. Immunogenicity of ZIKV M/E saRNA vaccine.

(A) *Ifnar1*$^{-/-}$ mice were treated as described in Figure 2A and analyzed on day 3 after retro-orbital challenge with 10^3 focus-forming units of ZIKV SD001. Data were collected from the same animal experiment shown in Figure 2D. qRT-PCR analysis of ZIKV RNA levels in the indicated organs. (B and C) Wild-type mice were treated as described in Figure 2C and analyzed on day 42 after receiving two doses of immunization. Data were collected from the same animal experiment shown in Figure 2F and G. Frequency (%) of IFNγ$^+$-producing,

polyfunctional ($IFN\gamma^{+}TNF\alpha^{+}$ or $IFN\gamma^{+}TNF\alpha^{+}IL\text{-}2^{+}$), or cytotoxic ($IFN\gamma^{+}CD107a^{+}$) $CD4^{+}$ (B) and $CD8^{+}$ (C) effector memory T cells ($CD3^{+}CD44^{+}CD62L^{-}$). Data are pooled from two independent experiments and are presented as the mean ± SEM. In panel A, for *Ifnar1*$^{-/-}$ mice, n=7 for the Rluc group and n=8 for the ZIKV M/E group. In panels B and C, for WT mice, n=10 per group. Circles represent individual mice. Dotted line indicates the limit of detection. *P < 0.05, **P < 0.01, ***P < 0.001 by the unpaired t-test.

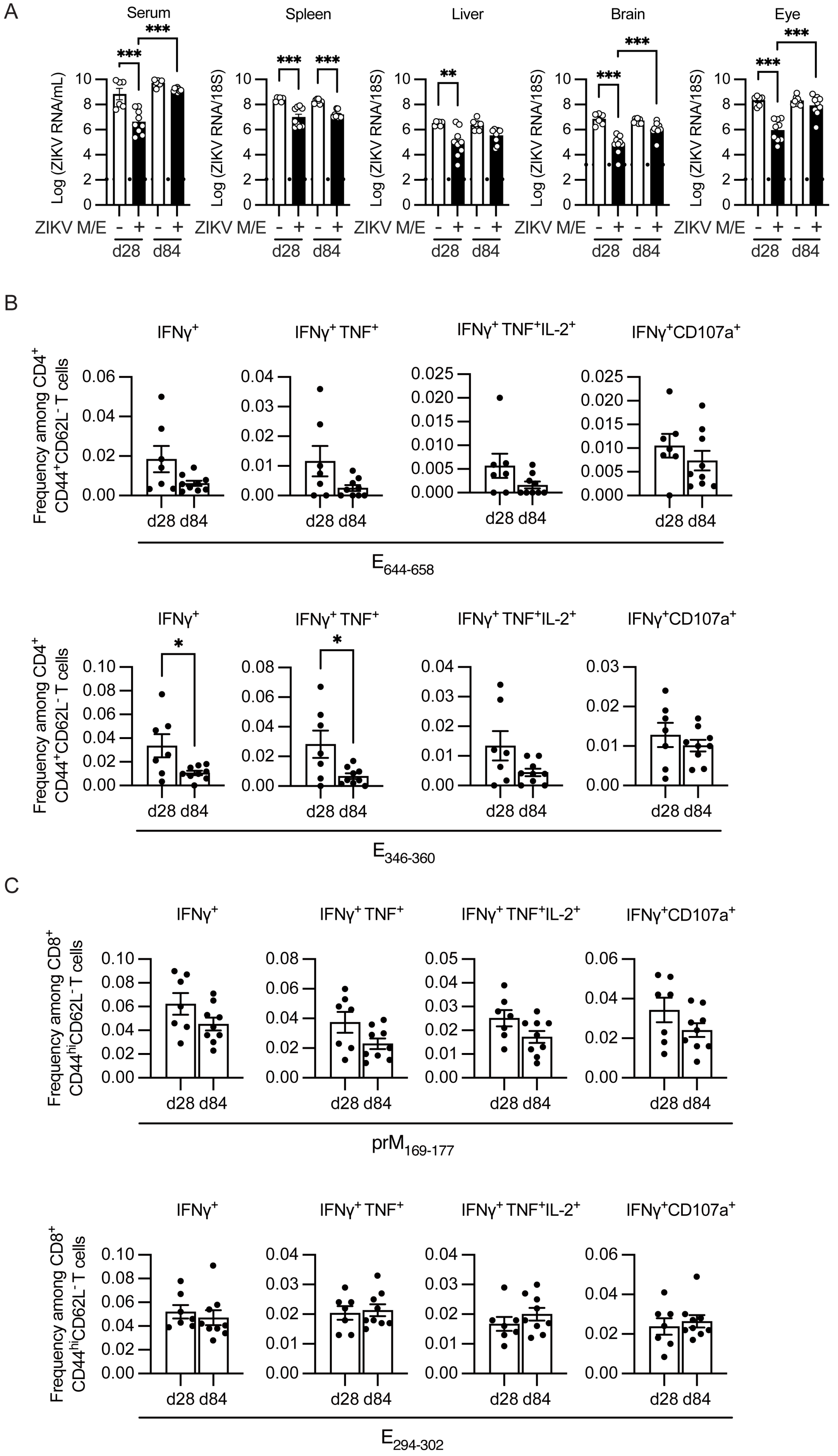

A
Serum
Spleen
Liver
Brain
Eye
Log (ZIKV RNA/mL)
Log (ZIKV RNA/18S)
ZIKV M/E
d28
d84
B
IFNγ+
IFNγ+ TNF+
IFNγ+ TNF+IL-2+
IFNγ+CD107a+
Frequency among CD4+ CD44+CD62L- T cells
E644-658
E346-360
C
Frequency among CD8+ CD44hiCD62L- T cells
prM169-177
E294-302

Fig S3. Short- and long-term immunogenicity and protective efficacy of ZIKV M/E saRNA vaccine.

(A) *Ifnar1*$^{-/-}$ mice were treated as described in Figure 3A and analyzed on day 3 after retro-orbital challenge with 10^6 focus-forming units of ZIKV SD001. Data were collected from the same animal experiment shown in Figure 3B. qRT-PCR analysis of ZIKV RNA levels in the indicated organs. (B and C) Wild-type (WT) mice were treated as described in Figure 3C and analyzed on either day 28 or day 84 after receiving one dose of immunization. Data were collected from the same animal experiment shown in Figure 3F and G. Frequency (%) of IFNγ^+-producing, polyfunctional (IFNγ^+TNFα^+ or IFNγ^+TNFα^+IL-2$^+$), and cytotoxic (IFNγ^+CD107a$^+$) CD4$^+$ (B) and CD8$^+$ (C) effector memory T cells (CD3$^+$CD4$^+$CD44$^+$CD62L$^-$) (see Figure S7 for gating strategy). Data are pooled from two independent experiments and are presented as the mean ± SEM. In panel A, for *Ifnar1*$^{-/-}$ mice, n=6 for the Rluc/d28 group, n=9 for the ZIKV M/E/d28 group, n=8 for the Rluc/d84 group, and n=10 for the ZIKV M/E/d84 group. In panels B and C, for WT mice, n=7 for the d28 group and n=9 for the d84 group. Circles represent individual mice. Dotted line indicates the limit of detection. $^*P < 0.05$, $^{**}P < 0.01$, $^{***}P < 0.001$ by one-way ANOVA with the Holm–Sidak multiple comparison test (A). $^*P < 0.05$ by the unpaired t-test (B and C).

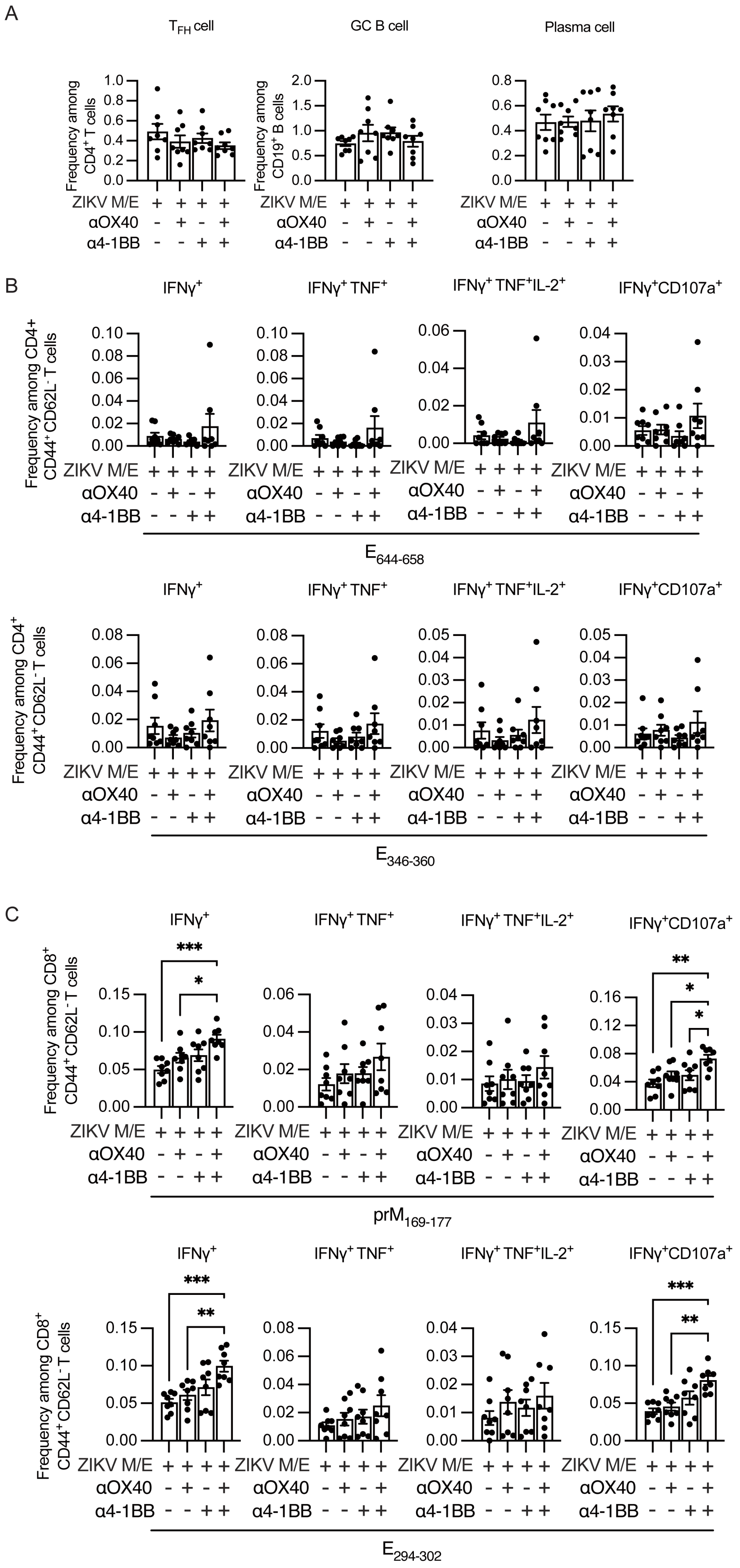

A
TFH cell
GC B cell
Plasma cell
Frequency among CD4+ T cells
Frequency among CD19+ B cells
ZIKV M/E
αOX40
α4-1BB
B
IFNγ+
IFNγ+ TNF+
IFNγ+ TNF+IL-2+
IFNγ+CD107a+
Frequency among CD4+ CD44+ CD62L- T cells
E644-658
E346-360
C
Frequency among CD8+ CD44+CD62L- T cells
prM169-177
E294-302

Fig S4. Effect of TNFR agonists on short-term immunogenicity.

Wild-type mice were treated as described for Figure 4A. Data were collected from the same animal experiment shown in Figure 4, D–F. (A) Frequency of T follicular helper (T_{FH}) cells ($CD3^+CD4^+CXCR5^+PD\text{-}1^+$), germinal center (GC) B cells ($CD19^+Fas^+GL7^+CD138^-IgD^-$), and plasma cells ($CD19^+CD138^+IgD^-$) in splenocyte populations. (B and C) Frequency of $IFN\gamma^+$-producing, polyfunctional ($IFN\gamma^+TNF\alpha^+$ or $IFN\gamma^+TNF\alpha^+IL\text{-}2^+$), or cytotoxic ($IFN\gamma^+CD107a^+$) $CD4^+$ (B) and $CD8^+$ (C) effector memory T cells ($CD3^+CD44^+CD62L^-$) among in vitro-stimulated splenocytes. Data are pooled from two independent experiments and are presented as the mean ± SEM of 8 mice/group in total. Circles represent individual mice. *$P < 0.05$, **$P < 0.01$, ***$P < 0.001$ by one-way ANOVA with the Holm–Sidak multiple comparison test.

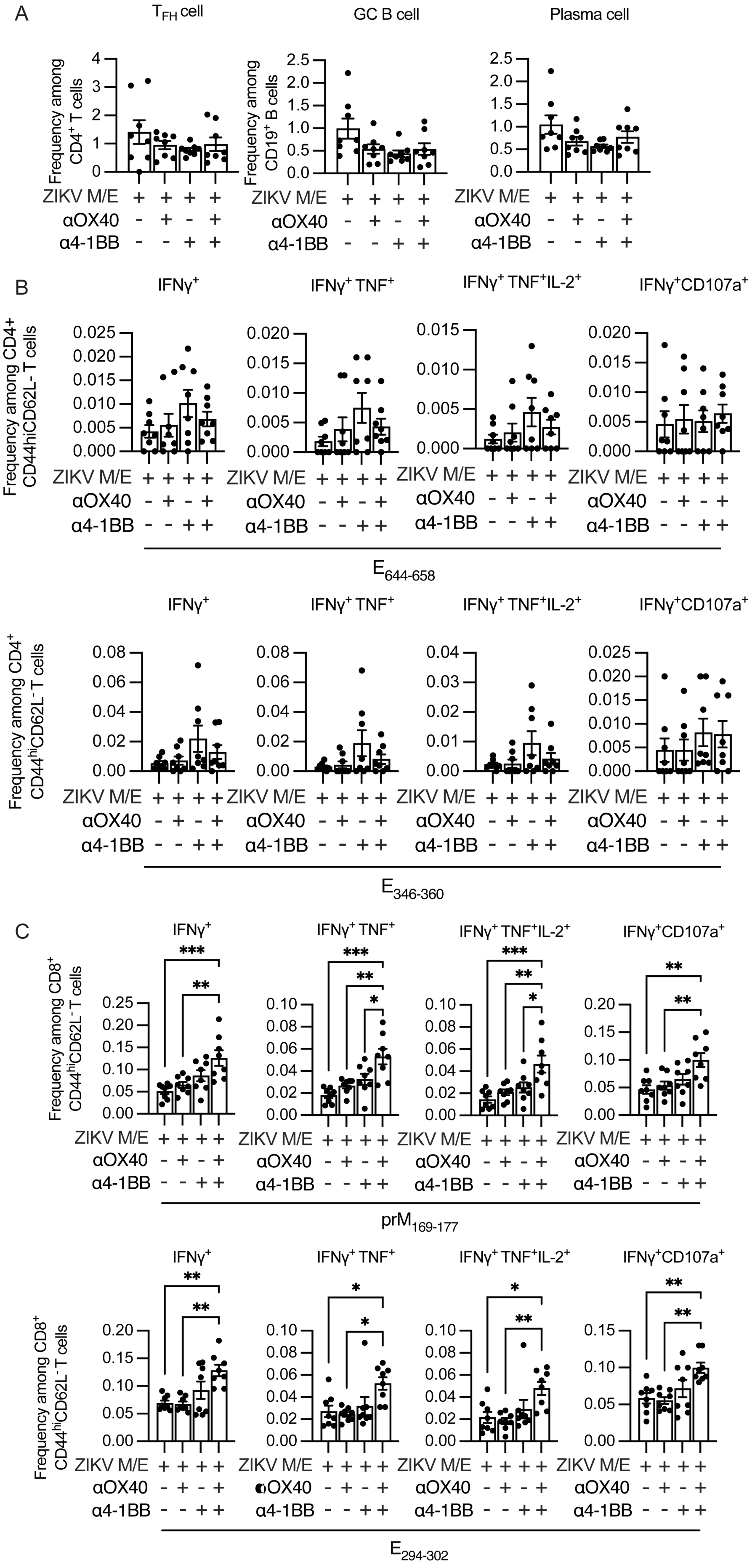
A
TFH cell
GC B cell
Plasma cell
Frequency among CD4+ T cells
Frequency among CD19+ B cells
ZIKV M/E
αOX40
α4-1BB
B
IFNγ+
IFNγ+ TNF+
IFNγ+ TNF+IL-2+
IFNγ+CD107a+
Frequency among CD4+ CD44hiCD62L- T cells
E644-658
E346-360
C
prM169-177
E294-302
Frequency among CD8+ CD44hiCD62L- T cells

Fig S5. Effect of TNFR agonists on long-term immunogenicity.

Wild-type mice were treated as described for Figure 5A. Data were collected from the same animal experiment shown in Figure 5, D–F. (A) Frequency (%) of T follicular helper (T_{FH}) cells ($CD3^+CD4^+CXCR5^+PD\text{-}1^+$), germinal center (GC) B cells ($CD19^+Fas^+GL7^+CD138^-IgD^-$), and plasma cells ($CD19^+CD138^+IgD^-$) in splenocyte populations. (B and C) Frequency of $IFN\gamma^+$-producing, polyfunctional ($IFN\gamma^+TNF\alpha^+$ or $IFN\gamma^+TNF\alpha^+IL\text{-}2^+$), or cytotoxic ($IFN\gamma^+CD107a^+$) $CD4^+$ (B) and $CD8^+$ (C) effector memory T cells ($CD3^+CD44^+CD62L^-$) among in vitro-stimulated splenocytes. Data are pooled from two independent experiments and are presented as the mean ± SEM of 8 mice/group in total. Circles represent individual mice. *P < 0.05, **P < 0.01, ***P < 0.001 by one-way ANOVA with the Holm–Sidak multiple comparison test.

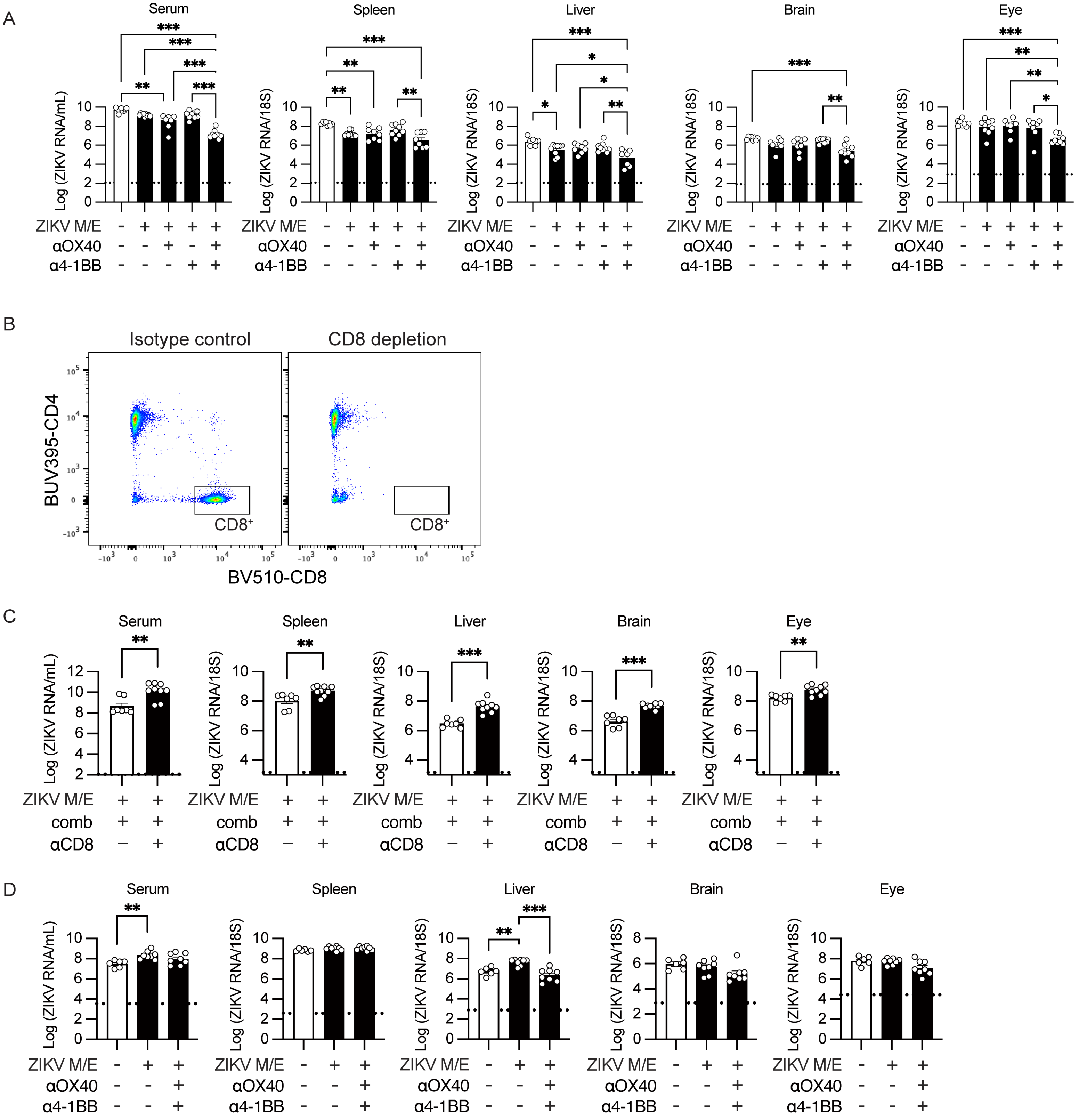

A
Serum
Spleen
Liver
Brain
Eye
Log (ZIKV RNA/mL)
Log (ZIKV RNA/18S)
ZIKV M/E
αOX40
α4-1BB
B
Isotype control
CD8 depletion
BUV395-CD4
BV510-CD8
CD8⁺
C
Serum
Spleen
Liver
Brain
Eye
Log (ZIKV RNA/mL)
Log (ZIKV RNA/18S)
ZIKV M/E
comb
αCD8
D
Serum
Spleen
Liver
Brain
Eye
Log (ZIKV RNA/mL)
Log (ZIKV RNA/18S)
ZIKV M/E
αOX40
α4-1BB

1005

Fig S6. CD8$^+$ T cell-driven long-term protection induced by OX40 and 4-1BB co-treatment

(A) *Ifnar1*$^{-/-}$ mice were treated as described in Figure 6A, and data were collected from the same animal experiment shown in Figure 6B. qRT-PCR analysis of ZIKV RNA levels in the indicated organs. (B and C) *Ifnar1*$^{-/-}$ mice were treated as described in Figure 6C, and data were collected from the same animal experiment shown in Figure 6D. (B) Flow cytometry analysis of CD8$^+$ T cell depletion efficiency, with the CD8$^+$ population gated on live CD3$^+$ cells. (C) qRT-PCR analysis of ZIKV RNA levels in the indicated organs. (D) *Ifnar1*$^{-/-}$ mice were treated as described in Figure 6E, and data were collected from the same animal experiment shown in Figure 6F. Data are pooled from two independent experiments and are presented as the mean ± SEM. In panel A, n=8 for the Rluc group, n=9 for the αOX40 or α4-1BB groups, and n= 8 for the αOX40/α4-1BB group. In panel C, n=7 for the isotype control group and n=9 for the αCD8 group. In panel D, n=6 for the Rluc group and n=8 for the isotype or αOX40/α4-1BB groups. Circles represent individual mice. Dotted line indicates the limit of detection. *P < 0.05, **P < 0.01, ***P < 0.001 by one-way ANOVA with the Holm–Sidak multiple comparison test (A and D). *P < 0.05, **P < 0.01, ***P < 0.001 by the unpaired t-test (C).

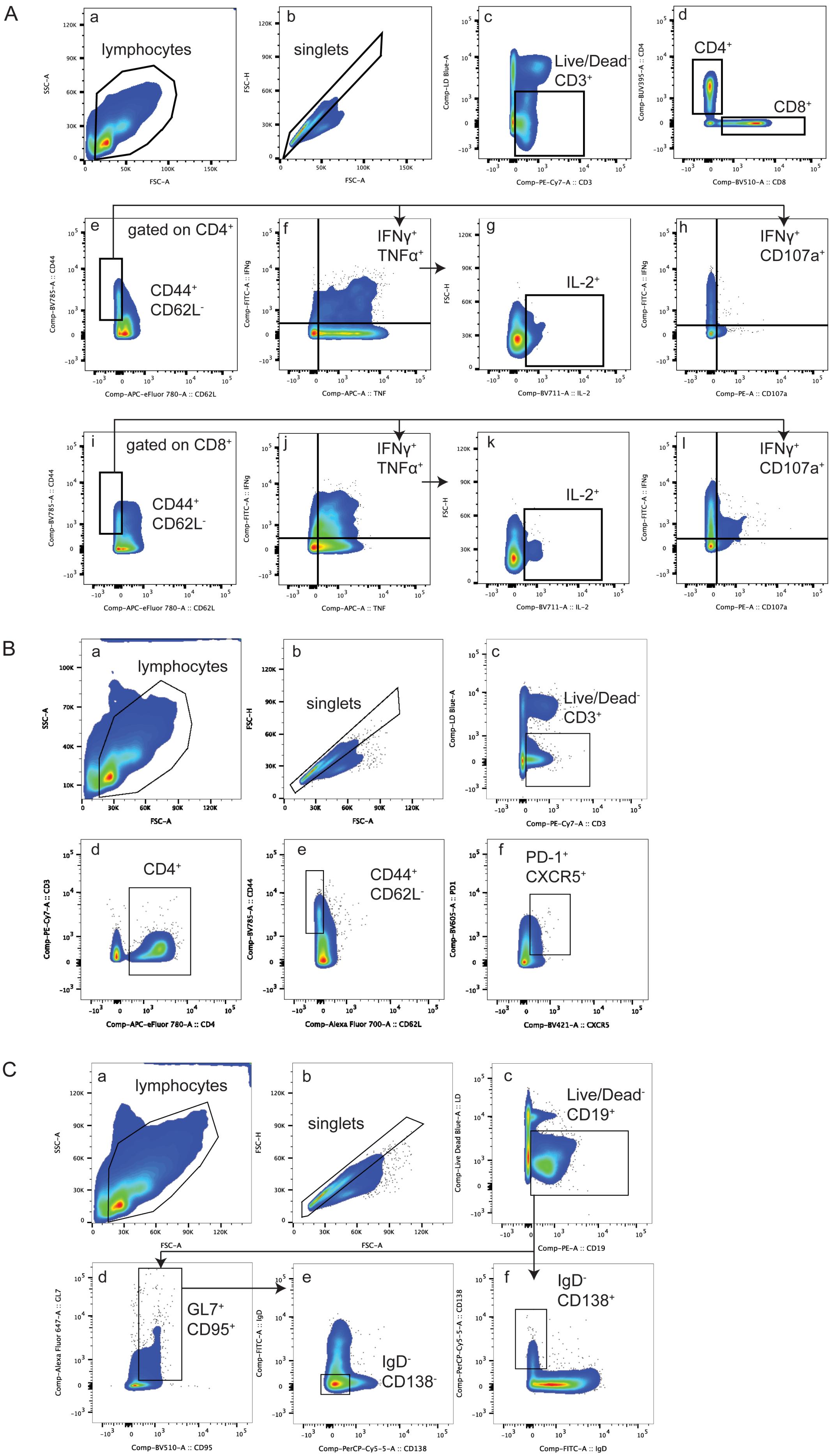
A
a
lymphocytes
b
singlets
c
Live/Dead-
CD3+
d
CD4+
CD8+
e
gated on CD4+
CD44+
CD62L-
f
IFNγ+
TNFα+
g
IL-2+
h
IFNγ+
CD107a+
i
gated on CD8+
CD44+
CD62L-
j
IFNγ+
TNFα+
k
IL-2+
l
IFNγ+
CD107a+
B
a
lymphocytes
b
singlets
c
Live/Dead-
CD3+
d
CD4+
e
CD44+
CD62L-
f
PD-1+
CXCR5+
C
a
lymphocytes
b
singlets
c
Live/Dead-
CD19+
d
GL7+
CD95+
e
IgD-
CD138-
f
IgD-
CD138+

Fig S7. Representative gating strategy.

(A) Gating strategy for the identification and enumeration of effector memory T cells (T_{EM}) (Live/Dead$^-$CD3$^+$CD4$^+$ [or CD8$^+$] CD44$^+$CD62L$^-$). The lymphocyte region was selected by size and granularity (FSC-A × SSC-A), followed by the exclusion of double events (b). Singlets were gated on viable CD3$^+$ cells (c), then for CD4$^+$ or CD8$^+$ T cells (d), and finally for effector memory cells (CD44$^+$CD62L$^-$) (e,i). Within the T_{EM} population, polyfunctional T cells were selected by expression of IFN-γ^+TNF-α^+ (f,j) or IFN-γ^+TNF-α^+IL-2$^+$ (g,k), and cytotoxic lymphocytes by expression of IFN-γ^+/CD107a$^+$ (h,l). (B) Gating strategy for the identification and enumeration of follicular helper T cells (T_{FH}) (Live/Dead$^-$CD3$^+$CD4$^+$CD44$^+$CD62L$^-$CXCR5$^+$PD-1$^+$ cells. (C) Gating strategy for the identification and enumeration of germinal center (GC) B cells (Live/Dead$^-$CD19$^+$Fas$^+$GL7$^+$CD138$^-$IgD$^-$ cells) and plasma cells (Live/Dead$^-$CD19$^+$CD138$^+$IgD$^-$ cells).
