# Supplementary material for: Enhanced durability of a Zika virus self-amplifying RNA vaccine through combinatorial OX40 and 4-1BB agonism
Source: JCI Insight. 2025 Apr 3;10(10):e187405. doi: 10.1172/jci.insight.187405 (PMC12128976; doi:10.1172/jci.insight.187405)

Full unedited gel for Figure 1B

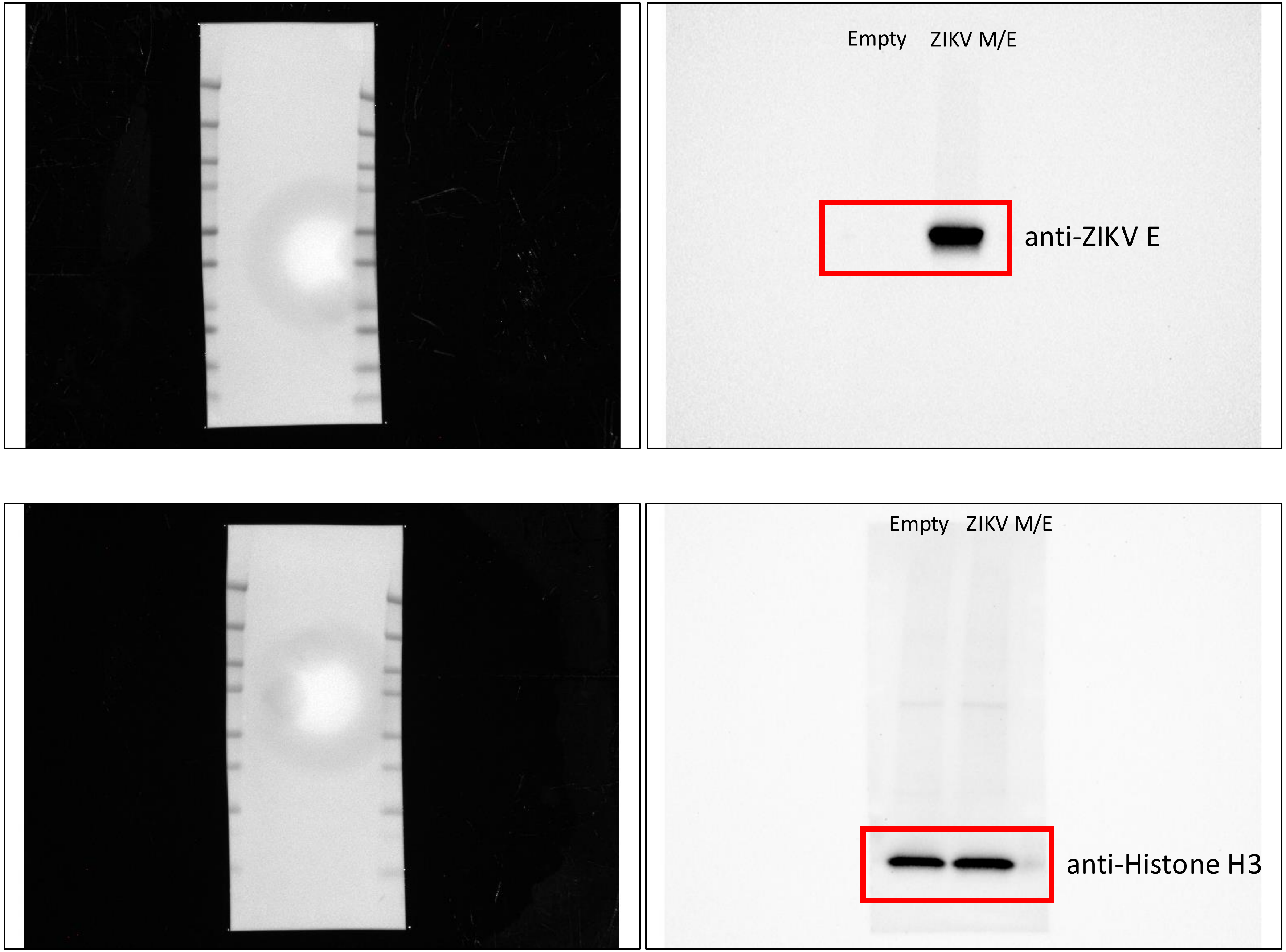

The membrane were first probed with anti-ZIKV E Ab. After imaging, the same membrane were stripped and re-probed with anti Histone H3 Ab.

Full unedited gel for Figure S1A

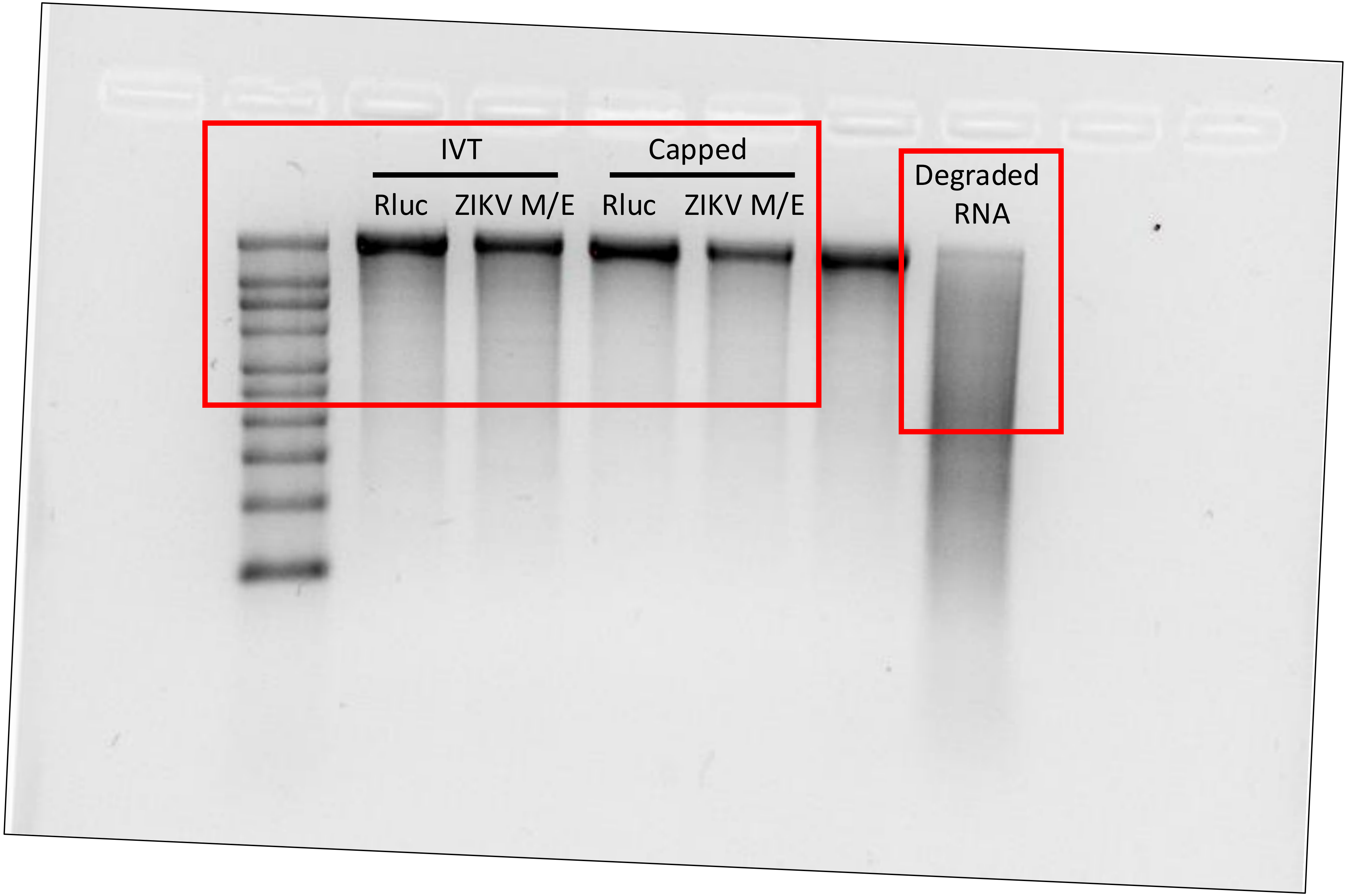

Supplement: Unedited blot and gel images [file jciinsight-10-187405-s069.pdf]
